# Real‐time Observation of Structural Dynamics Triggering Excimer Formation in a Perylene Bisimide Folda‐dimer by Ultrafast Time‐Domain Raman Spectroscopy

**DOI:** 10.1002/anie.202114474

**Published:** 2022-02-07

**Authors:** Yongseok Hong, Woojae Kim, Taeyeon Kim, Christina Kaufmann, Hyungjun Kim, Frank Würthner, Dongho Kim

**Affiliations:** ^1^ Department of Chemistry Spectroscopy Laboratory for Functional π-Electronic Systems Yonsei University 03722 Seoul Republic of Korea; ^2^ Institut für Organische Chemie & Center for Nanosystems Chemistry Universitat Würzburg Am Hubland 97074 Würzburg Germany; ^3^ Department of Chemistry Incheon National University 119 Academy-ro, Yeonsu-gu 22012 Incheon Republic of Korea; ^4^ Department of Chemistry and Chemical Biology Cornell University Ithaca 14853 New York USA; ^5^ The Institute for Sustainability and Energy at Northwestern Northwestern University Evanston 60208 Illinois USA

**Keywords:** Excimer, Perylene Bisimide, Structural Dynamics, Time-Resolved Impulsive Stimulated Raman Spectroscopy, Vibrational Coherence

## Abstract

In π‐conjugated organic photovoltaic materials, an excimer state has been generally regarded as a trap state which hinders efficient excitation energy transport. But despite wide investigations of the excimer for overcoming the undesirable energy loss, the understanding of the relationship between the structure of the excimer in stacked organic compounds and its properties remains elusive. Here, we present the landscape of structural dynamics from the excimer formation to its relaxation in a co‐facially stacked archetypical perylene bisimide folda‐dimer using ultrafast time‐domain Raman spectroscopy. We directly captured vibrational snapshots illustrating the ultrafast structural evolution triggering the excimer formation along the interchromophore coordinate on the complex excited‐state potential surfaces and following evolution into a relaxed excimer state. Not only does this work showcase the ultrafast structural dynamics necessary for the excimer formation and control of excimer characteristics but also provides important criteria for designing the π‐conjugated organic molecules.

## Graphical abstract

The term “excimer”, a contraction of an excited dimer, is the most classical photoproduct in organic materials. In this work, vibrational snapshots showing ultrafast structural dynamics triggering the excimer formation and its relaxation are presented based on ultrafast time‐domain Raman spectroscopy. It is demonstrated that interchromophore vibrational modes are related to perturbing the excited‐state potential surface of a dimer stack.

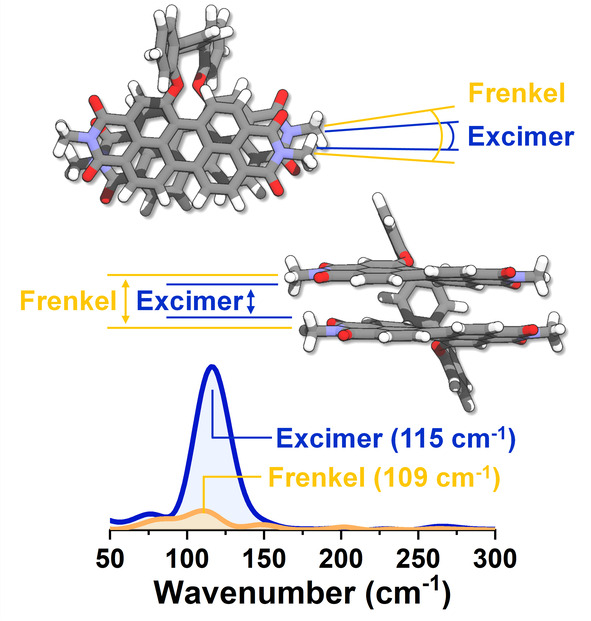

## Introduction

An excimer state has often been observed upon photoexcitation of π‐conjugated organic materials, which are widely used in the field of organic photovoltaics.[^1^, ^2^, ^3^, ^4^, ^5^, ^6^, ^7^, ^8^, ^9^, ^10^, ^11^, ^12^, ^13^] While the excimer classically meant a dimerization of the same species in the excited state, the concept has been expanded into the dimers or oligomers, which are already electronically coupled in the ground state. Considering that the excimer state is typically observed in the solid phase where π–π distance lies between 3 to 5 Å,[^8^, ^9^, ^10^, ^11^] performance in such photovoltaic devices has been limited due to the excimer state acting as a low energy trap state which results in a large activation energy barrier toward efficient energy and charge transport.[^2^, ^3^, ^4^, ^5^, ^6^, ^7^] To avoid undesired energy loss by the trap state, it is crucial to understand how the excimer state is formed, and its properties are changed. Since the intermolecular interactions such as Coulomb (long‐range) interaction and charge‐transfer (CT) mediated superexchange (short‐range) interaction are hyper‐sensitive to molecular packing structures,[^10^, ^11^] it has been suggested that morphological control is crucial for inducing the charge‐separated (CS)^[12]^ or multiexcitonic (ME)[^12^, ^13^] intermediate states. However, it is difficult to generalize the molecular design principles for the desirable photovoltaic devices through synthetic approaches due to the complexity of the excited state.

In this regard, researchers have theoretically and experimentally investigated the nature of the excimer state from its formation to relaxation processes in π‐stacked molecular assemblies. As for theoretical studies,[^14^, ^15^, ^16^, ^17^] it has been suggested that the shortening of intermolecular distance and changes in translational or rotational displacements between monomer units toward perfect sandwich geometry perturb the electronic structures in the excited state and provide significant stabilization energy, finally leading to the excimer formation.^[17]^ Experimentally, various time‐resolved electronic spectroscopies revealed that the excimer is formed within a sub‐picosecond to a few picosecond timescales accompanied by the evolution of vibronic peak ratio in transient fluorescence spectra and the rise of characteristic near‐infrared (NIR) absorption band of the excimer in femtosecond transient absorption (fs‐TA) spectra.[^18^, ^19^] Beyond the excimer formation, researchers have recently suggested that the nature of the excimer state could be modulated, depending on the local environment as well as conformational control, towards a symmetry‐breaking (SB) CS state,[^20^, ^21^, ^22^] charge resonance (CR) enhanced excimer state,^[23]^ or ME state^[24]^ by controlling the CT character of the excimer state. These infer the potentials of the excimer state to be applicable for organic photovoltaic (OPV) devices.

However, in contrast to the wealth of theoretical and experimental studies, the direct observation of the ultrafast structural dynamics triggering excimer formation has remained elusive so far. In previous works, several investigations on the structural information of the excimer state have been reported using femtosecond stimulated Raman spectroscopy (FSRS),^[25]^ time‐resolved infrared spectroscopy (TR‐IR),[^26^, ^27^, ^28^] and TA spectroscopy.[^29^, ^30^, ^31^, ^32^] However, these techniques have revealed some technical limits, such as non‐trivial background signals (FSRS) and a lack of target selectivity (TR‐IR and TA). On the one hand, our group has very recently reported the characteristic intermolecular low‐frequency vibrations of the excimer state in cofacially stacked one‐dimensional perylene bisimide (PBI) H‐aggregates by using ultrafast time‐domain Raman spectroscopy.^[33]^ We have suggested this low‐frequency interchromophore vibration presumably plays an important role in the excimer formation, however, have not analyzed the early‐time dynamics in detail. Therefore, it has remained still challenging to characterize full structural dynamics, which is of utmost importance to unveil how the excimer state is formed in the context of manipulating ultrafast photophysics of π‐stacked organic materials.

**Figure 1:**
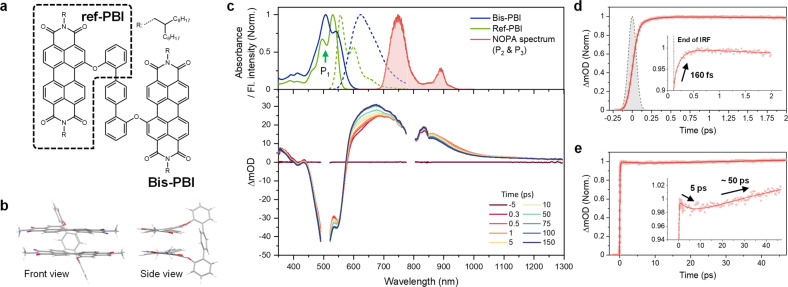
a) A schematic molecular structure of **Bis‐PBI** and its monomeric counterpart, **ref‐PBI** (the box with a dashed line). b) Front and side views of the ground‐state‐optimized structure (*ω*B97X‐D/6‐31g(d)) of **Bis‐PBI**. The alkyl chains substituted at imide positions are replaced by methyl groups to reduce computational costs. c) Normalized steady‐state absorption (solid line) and fluorescence (dashed line) spectra (top) of **Bis‐PBI** (blue) and **ref‐PBI** (green) in toluene. The green arrow at 510 nm indicates wavelength for actinic pump (P_1_) in TA and TR‐ISRS experiments. The NOPA spectrum (red shaded) for Raman pump (P_2_) and probe (P_3_) in TR‐ISRS experiment is also shown. TA spectra of **Bis‐PBI** in toluene at certain delay times (bottom) measured with a Ti:Sapphire system (200–300 fs time resolution). Full spectral evolution up to 1 ns is shown in Figure S6. TA kinetics of **Bis‐PBI** in toluene up to (d) 2 ps and (e) 48 ps measured by P_1_ and P_3_ pulses with a Yb : KGW system. The dashed line in panel (d) indicates instrument response function (IRF) of the system (120 fs). The red solid line corresponds to the best‐fitted curve convolved with IRF and multiexponential functions. Insets show an enlarged view of the kinetics, revealing complex multiexponential changes of the dynamics from femtosecond to picosecond regimes.

To decipher the details of full structural dynamics in excimer formation, which has not been experimentally revealed, we herein have prepared an archetypical PBI folda‐dimer, **Bis‐PBI** (Figure 1a), where two PBIs are tethered by a terphenyl spacer through bay positions.^[34]^ **Bis‐PBI** can be regarded as one of the simplest H‐aggregates so that we can rule out intimate conformational heterogeneity derived from long‐range molecular aggregates and resulting in complicated exciton dynamics.[^13^, ^34^] Furthermore, we have utilized time‐resolved impulsive stimulated Raman spectroscopy (TR‐ISRS),[^35^, ^36^, ^37^, ^38^, ^39^, ^40^, ^41^, ^42^, ^43^] allowing us to capture vibrational snapshots at various time delays after photoexcitation, which includes the information on complete structural evolution from the initial Frenkel‐like exciton to the excimer state. Our time‐resolved Raman results prove that structural changes via the specific interchromophore vibrational coordinates, which have been theoretically suggested, actually pertain to the fate and nature of the excimer state.

## Results and Discussion

In toluene, (TOL, *ϵ*=2.38), while the absorption spectrum of reference PBI monomer (**ref‐PBI**, Figure 1a) shows well‐resolved vibronic progression with the vibronic peak ratio between 0–0 and 0–1 transition (*A*_0‐0_/*A*_0‐1_) of ≈1.56, the absorption spectrum of **Bis‐PBI** (Figure 1a) reveals a distinct inversion of *A*_0‐0_/*A*_0‐1_ to ≈0.75 (Figure 1c, top). This clearly indicates intermolecular interactions arising from the co‐facial geometry of the dimer, which is in line with the expected structure modeled by density function theory (DFT) calculations (Figure 1b).^[34]^ Borrowing the interpretation from Spano's expanded Kasha theory,[^10^, ^11^] **Bis‐PBI** belongs to a Hj‐type dimer, which shows large Coulombic but small CT coupling. The fluorescence spectrum becomes significantly broader and red‐shifted and also the fluorescence quantum yield decreases compared to **ref‐PBI** (Figure S4 and Table S1), confirming that the relaxed excited state is the excimer state. Furthermore, **Bis‐PBI** exclusively shows red‐shift in fluorescence spectra and decrease in quantum yields, as the solvent polarity increases. This indicates that the dipolar solvation process stabilizes the energy of the emitting excimer state and opens more efficient non‐radiative deactivation pathways, which is similar to recently reported CR‐enhanced excimer.^[23]^ Overall, these results underpin that the excimer state can be viewed as an adiabatic mixture of locally excited (LE) and CR configurations due to the energetic proximity of the two diabats or strong coupling.[^10^, ^23^, ^44^]

To understand the excited‐state dynamics of **Bis‐PBI**, we first carried out fs‐TA experiments with two different laser systems (a Ti:Sapphire system for dispersed detection with CMOS cameras and a Yb:KGW system for non‐dispersed detection with photodiodes, see Supporting Information). With the dispersed detection, we have measured TA spectra from 350 nm to 1300 nm. In TOL, excluding the ground‐state bleach (GSB) contributions, **Bis‐PBI** only shows broad excited‐state absorption (ESA) bands in the range of 600–1300 nm without stimulated emission signals due to weak emitting properties (Figure 1c, bottom), which is highly similar to those of other PBI stacks relaxing to the excimer state.[^18^, ^20^, ^23^] These spectral characteristics maintain consistency from the initial (0.3 ps) to later (1 ns) delay time, whereas significant rise and decay dynamics were observed in 580–850 nm and 850–1300 nm regions, respectively. Both regions reveal biexponential kinetics of 3–5 ps and 40–120 ps (Figure S6), implying multiphasic relaxation processes of photoexcited **Bis‐PBI**. The slowest component, >10 ns, corresponds to excimer‐state lifetime, which is in good agreement with the data previously measured by the TCSPC method.[^13^, ^34^] Especially, by the non‐dispersed detection in the 700–950 nm range, with the aid of the faster time‐resolution (120 fs) of the system, we could additionally capture an ultrafast rise component of ≈160 fs (Figure 1d). This time constant can be attributed to the excimer formation based on our previous observation on various PBI assemblies with femtosecond time‐resolved fluorescence and absorption techniques.[^18^, ^45^] Additional biexponential kinetics of 5 and 50 ps were also revealed (Figure 1e), analogous to the dispersed‐detection‐based results (Figure S6).

In polar environments (tetrahydrofuran, THF, *ϵ*=7.58; dichloromethane, DCM, *ϵ*=8.93; Benzonitrile, BCN, *ϵ*=26.0), the initial TA spectra at 0.3 ps reveal a similar shape compared to that in TOL (Figure S7). On the other hand, as the relaxation progresses, ESA bands at 600 nm and 720 nm become relatively sharper. Considering that those pertain to cation and anion bands of **ref‐PBI** but maintain broader shapes, this result implies that CR configuration of the excimer state can be enhanced through incomplete SB‐CS by solvation processes (like exciplexes) in polar media.[^23^, ^46^, ^47^] Furthermore, in a highly viscous TOL:paraffin oil mixture (1:9, v:v), both the amplitudes and time constants for the relaxation processes become considerably lower, suggesting that the picosecond‐order relaxation is also related to large‐amplitude structural motion towards the relaxed excimer geometry. Especially for the TR‐ISRS experiments, we have chosen TOL and THF to track the structural evolution along with the excimer formation and relaxation dynamics based on their different polarity but similar viscosity.

**Figure 2:**
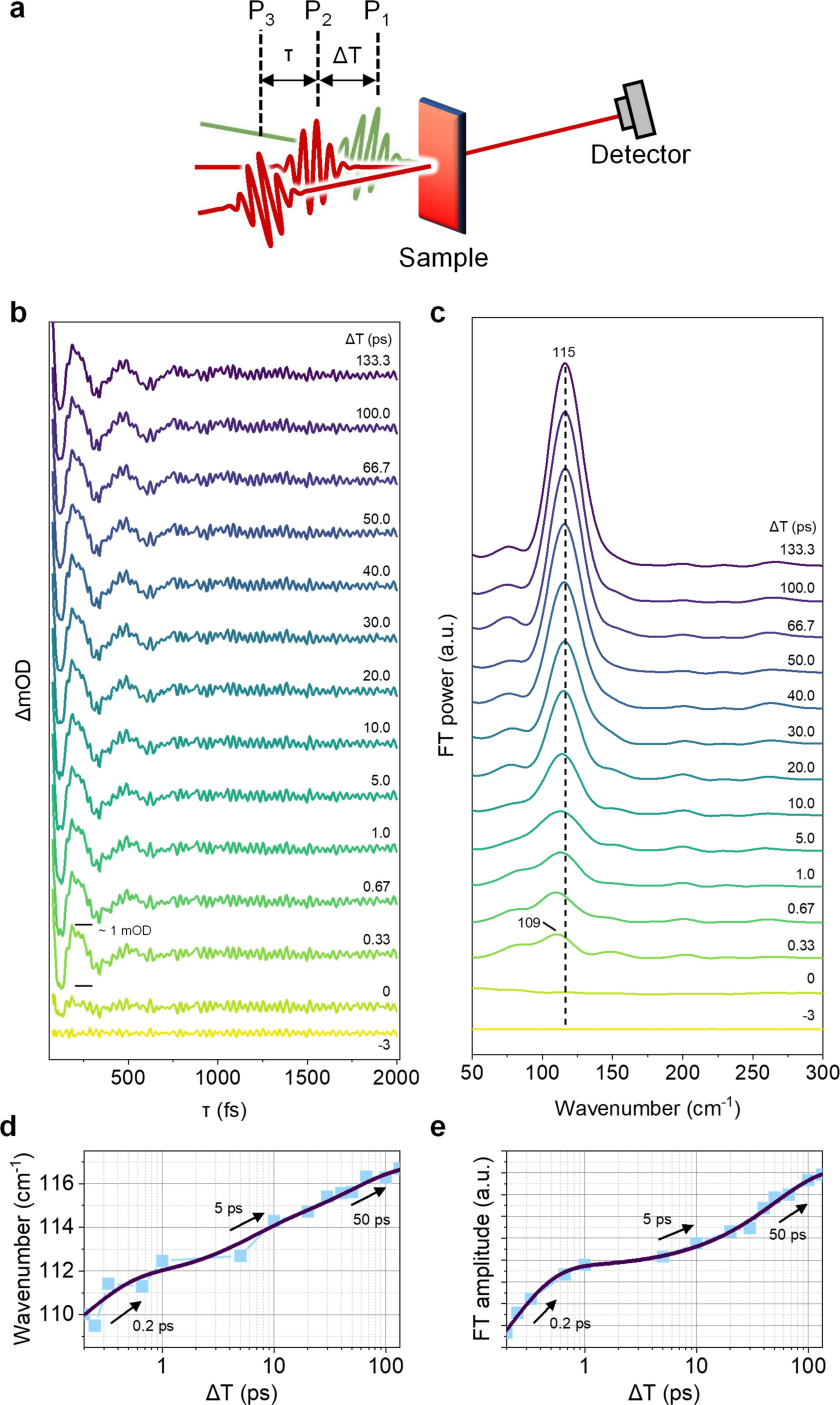
a) Schematic illustration of the TR‐ISRS experiment. b) Time‐domain oscillatory signals obtained from the raw TR‐ISRS signals (Figure S10) of **Bis‐PBI** in TOL. c) FT power spectra at various Δ*T* delay times. A dashed line is a guide to the eye to highlight the time‐dependent blue‐shift of the xOOP mode. Time‐dependent (d) frequency shift and (e) FT amplitude kinetics of the xOOP mode. Peak positions and FT amplitudes are estimated from Gaussian fits.

To elucidate the full structural changes during the excimer formation and relaxation, TR‐ISRS was conducted (Figures 2 and S8 and S9). As in the TA experiments, an actinic pump (P_1_) of 510 nm generates the excited state population. In TR‐ISRS, subsequent near‐infrared Raman pump (P_2_, sub‐10 fs) is resonant with the broad ESA band of the excimer state in the range of 700–950 nm,[^20^, ^23^] after an arbitrary time delay of Δ*T*. This triggers the vibrational coherence (VC) in the excited state by the resonant ISRS (here, the last S denotes “scattering”) process.^[48]^ Then, the Raman probe pulse (P_3_, sub‐10 fs), the replica of P_2_, records P_2_‐induced differential absorption signals with a time delay τ. The excited‐state Raman spectra are obtained by Fourier transformation (FT) of oscillatory residuals (Figure 2, panels b and c) extracted by subtraction of population kinetics with multiexponential fitting. Note that post‐processing procedures, such as zero padding and apodization, were used to improve the spectral quality.

The time‐resolved excited‐state Raman spectra in the low‐frequency region (50–300 cm^−1^) are shown in Figure 2c. In the high frequency region, we could find several Raman modes but their signal intensities are much lower (Figure S9), which are presumably due to the following two reasons: 1) decreased vibronic coupling strengths for high‐frequency modes in the dimer due to electronic delocalization,^[49]^ and/or 2) an absence of resonance enhancement (i.e., Albrecht *A* term) because of no displacement changes upon the lowest excited‐ to the higher excited state transition along those vibrational coordinates.^[50]^ Therefore, we only focused our investigation on the vibrational dynamics in the low‐frequency region. To discuss the structural dynamics accompanying the excimer formation and relaxation, we first extracted the peak positions of specific Raman modes from the FT power spectra. There are no sample signals observed at −3 ps (only solvent Raman modes are revealed, which is presumably due to a non‐flat sensitivity of the photodiode detector or a color filter effect arising from P_1_‐induced transient absorption signals),^[39]^ whereas a few Raman bands rapidly increase after time zero, confirming that these modes purely originate from photoexcited **Bis‐PBI**. A closer look at the evolution of the excited‐state Raman spectra reveals that the Raman mode at 109 cm^−1^ initially appears and immediately shows the frequency blue‐shift of about 3 cm^−1^ within 1 ps (Figure 2d). The time constant of 200 fs for the frequency blue‐shift of this low‐frequency mode and its excellent agreement with the TA kinetics strongly suggest that initial structural changes through this vibrational coordinate can be regarded as a main trigger for the excimer formation. After the excimer formation, the interchromophore out‐of‐plane (xOOP) mode shows further frequency shift with 5 and 50 ps time constants (Figure 2d), which could be assigned to the additional structural relaxation process towards a potential minimum of the excimer state, as observed in the TA kinetics. Also, in polar THF, the same frequency blue‐shift of 200 fs and ensuing relaxation of tens of picoseconds for the xOOP mode were identically revealed (Figure S13), signifying a negligible role of dipolar solvation in excimer formation of **Bis‐PBI**. Overall, the time‐dependent evolution of the xOOP mode strongly proposes that an interchromophore coordinate between the two neighboring PBIs is intimately linked with the excimer formation and relaxation. In contrast, the relaxation dynamics of xOOP mode were accelerated by more than five times in THF compared to weak polar TOL with the help of the dipolar solvation process. This result unambiguously reflects that the external environment affects the structural dynamics as well as the change of the excimer characteristics during the excimer relaxation. Moreover, it is intriguing that the other vibrational modes at 480 and 530 cm^−1^ in THF exclusively respond to the excimer relaxation processes. Although it is unclear that these vibrational modes directly induce further evolution into the CR‐enhanced excimer state, it is manifested that the dipolar solvation process gives rise to the prominent appearance of these modes. Therefore, we suggest that a few vibrational modes could be associated with the CR‐increasing reaction in the excimer state.

The additional two‐color TA experiment with sub‐10 fs visible pump reveals a similar mode at 100 cm^−1^ in the GSB (590—630 nm) region in the wavelength‐dependent FT power map (Figure S14). DFT calculations in the ground state of **Bis‐PBI** and allow us to tentatively assign this mode as an interchromophore out‐of‐plane (xOOP) vibrational mode that mainly modulates interchromophore (between two PBIs) distance and rotational angle simultaneously (see Figure 5b).^[13]^ On the other hand, in the ESA region (700—900 nm), this mode is slightly blue shifted, appearing at around 110 cm^−1^. This is a similar value compared to the TR‐ISRS data, indicating its origin from the excited state. The relatively higher value of the excited‐state VC compared to the ground‐state one suggests structural changes along its vibrational coordinate. In addition, sliding window FT with this mode in the ESA region exhibits a gradual blue‐shift with the time constant of approximately 200 fs,^[52]^ which underpins that the frequency evolution of the xOOP mode in the TR‐ISRS experiments represents the structural dynamics from the initial Frenkel‐like exciton near the Franck‐Condon region (Figure S14g).

**Figure 3:**
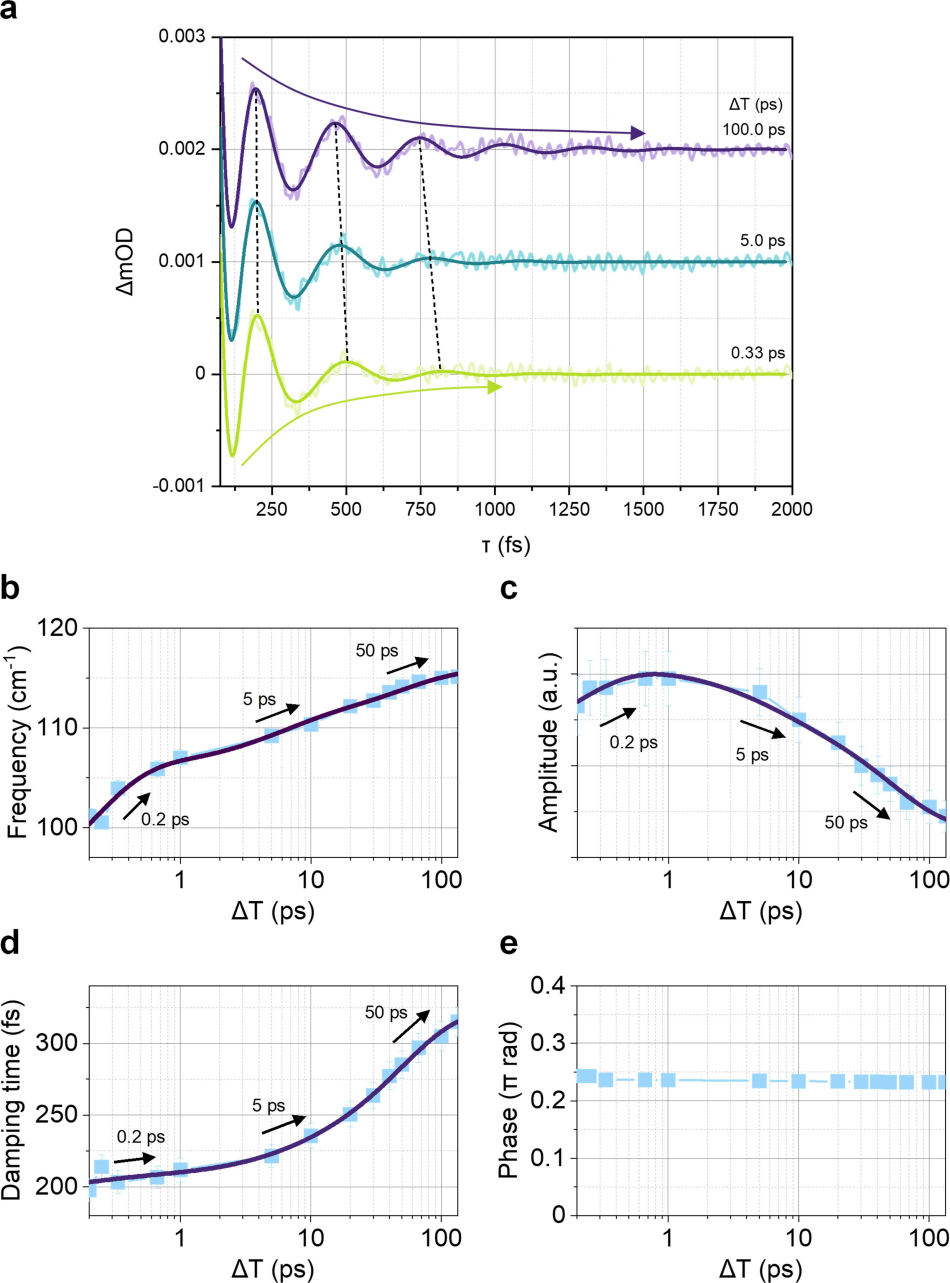
a) Representative damped sinusoidal fit curves (solid lines) at different Δ*T* delay times. Colored arrows and dashed black lines highlight Δ*T*‐dependent damping times and modulation of oscillating frequencies, respectively. Fit results depending on Δ*T*: b) frequency, c) amplitude, d) damping time, and e) phase.

In addition to frequency shifts, it is evident that only the xOOP mode reveals dramatic and continuous increases in FT amplitude (Figure 2e), further suggesting its relevance to the excimer formation and relaxation processes. This amplitude rise also reveals similar time constants compared to the frequency shift dynamics, which implies their same physical origin. However, here we have to consider three factors that can contribute to the amplitude rise dynamics in the frequency domain data: 1) an increase in ESA signals around wavelength region covered by the Raman pump and probe 2) time‐dependent displacement changes through a certain vibrational coordinate. And 3) an increase in a damping time of VC, which causes bandwidth narrowing and amplitude enhancement. Among the three factors, the first two bring about an increase in amplitudes of VC itself in the time domain through resonance enhancement and the last one is related to the mathematical effect. In particular, apodization, which we used to enhance the spectral quality, applied in the time‐domain data before FT can interrupt the analysis in terms of the third factor. In this sense, to discriminate the main effect, we have additionally analyzed the raw time‐domain data without any post‐processing by using a sum of damped sinusoidal functions to get information on amplitudes, dephasing times, frequencies, and phases of the VCs (Figure 3).

To properly fit long‐period oscillations mainly shown in the time‐domain data, two frequencies, ≈110 and 150 cm^−1^, were needed and the detailed fit parameters are described in Table S2. First, as for 150 cm^−1^, it dephases with 40 fs, is Δ*T* independent, and is not our interest even in the frequency domain data so we did not analyze it in detail. In terms of 110 cm^−1^, i.e., the xOOP mode, as in the frequency‐domain analysis, the oscillation frequency reveals blue‐shifts from ≈101 to ≈115 cm^−1^ with the time constants of about 0.2, 5, and 50 ps. The extent of frequency shift in the time‐domain analysis is slightly more significant than that in the frequency domain one but the consistency of time constants between the two allows us to support the validity of our analysis. The VC amplitude of the xOOP mode initially increases with the time constant of 200 fs but subsequently decreases biexponentially with 5 and 50 ps. Both time‐and frequency‐domain analyses imply that the continuous increases in FT amplitude does not originate from the population dynamics. Interestingly, we found the increase in dephasing time of xOOP mode VC as a function of Δ*T*. When VC of the xOOP mode is generated at earlier Δ*T* within 1 ps, it dephases with the time constant of approximately 200 fs, which is a similar value compared to the excimer formation rate. In sharp contrast, at later Δ*T* when excimer is formed, a dephasing time is elongated up to about 320 fs (at Δ*T*=100 ps). This signifies that the origin of amplitude rises of the xOOP mode shown in Figure 2 stems from the noticeable and continuous growth (in an increasing direction) in dephasing times of its VC depending on Δ*T*. Furthermore, it provides deeper insights into the reaction mechanism of the excimer formation. Typically, vibrational dephasing in the excited state can be affected by several factors, such as 1) population decay of the state where VCs are generated,^[53]^ 2) vibrational relaxation (vibrational cooling or intramolecular vibrational redistribution),^[51]^ 3) (an)harmonicity of the potential^[60]^, 4) vibronic coupling of the reaction.^[55]^ Here, the first factor can easily be ruled out, because excimer formation is not simple population kinetics between the two diabatic states and also not as an internal conversion (see below), for instance, from the S_2_ to S_1_ states. We propose that vibronic coupling can play a primary role in the ultrafast excimer formation, which means that the xOOP mode acts as a promotor mode. A piece of evidence is the similar time constants between VC damping (at early Δ*T*) and excimer formation. Another evidence is Δ*T*‐dependent damping times, suggesting that the xOOP mode is not a mere spectator mode. In other words, VC rapidly dephases since structures continuously evolve along the xOOP coordinate. This is a similar picture of the nested free energy crossing model in ultrafast electron transfer reaction, experimentally reported recently using ultrafast TA.[^54^, ^55^] After all structural changes are finished, the possible factors that can determine the dephasing time are vibrational relaxation and anharmonicity. Especially, in order to investigate the effect of anharmonicity, a two‐dimensional Raman technique is required to get information about coupling between vibrations, ^[61,62]^ but this is not the main interest in this work and will be investigated in the future.

**Figure 4:**
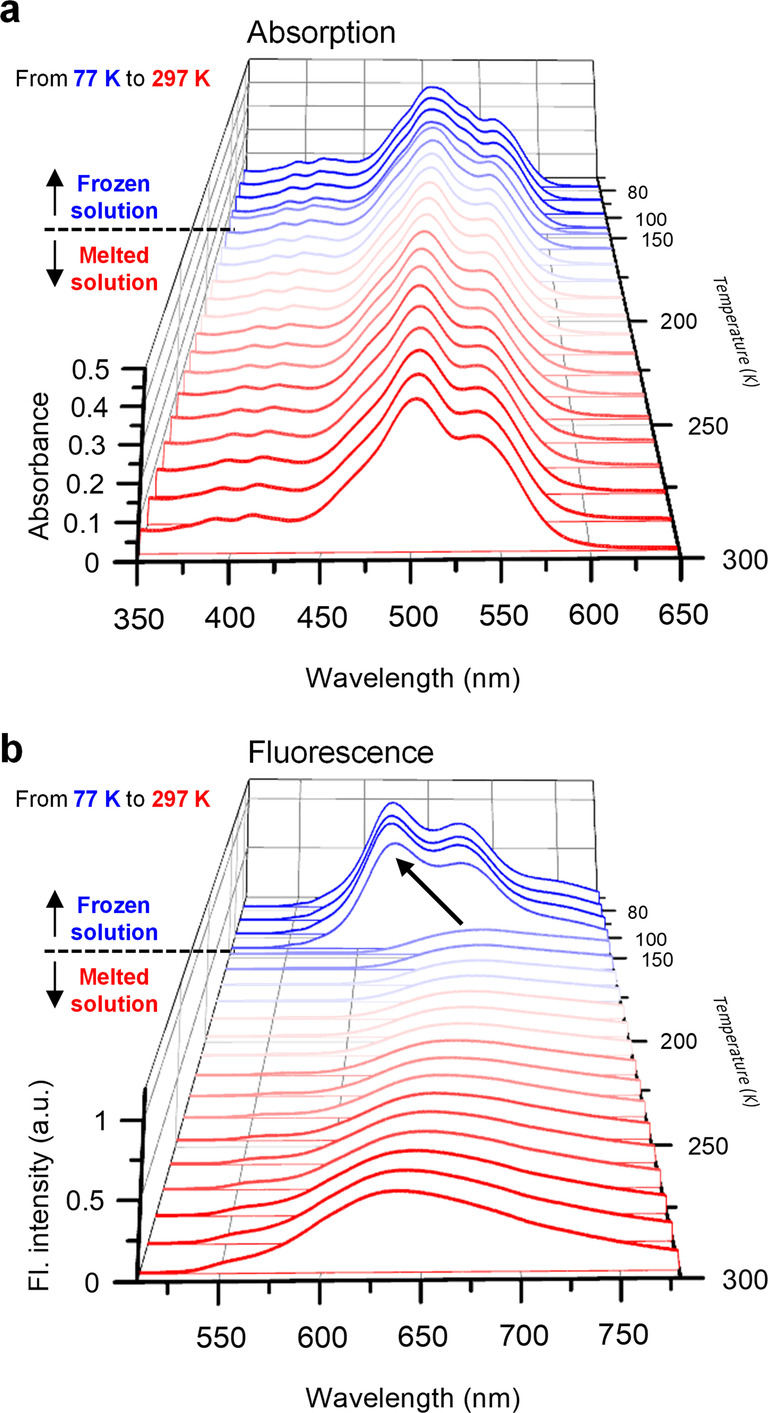
Temperature‐dependent steady‐state absorption (a) and fluorescence spectra (b) of **Bis‐PBI** in 2‐MeTHF. A black arrow in panel (b) indicates a sudden jump of fluorescence spectra near the melting point of 2‐MeTHF.

**Figure 5:**
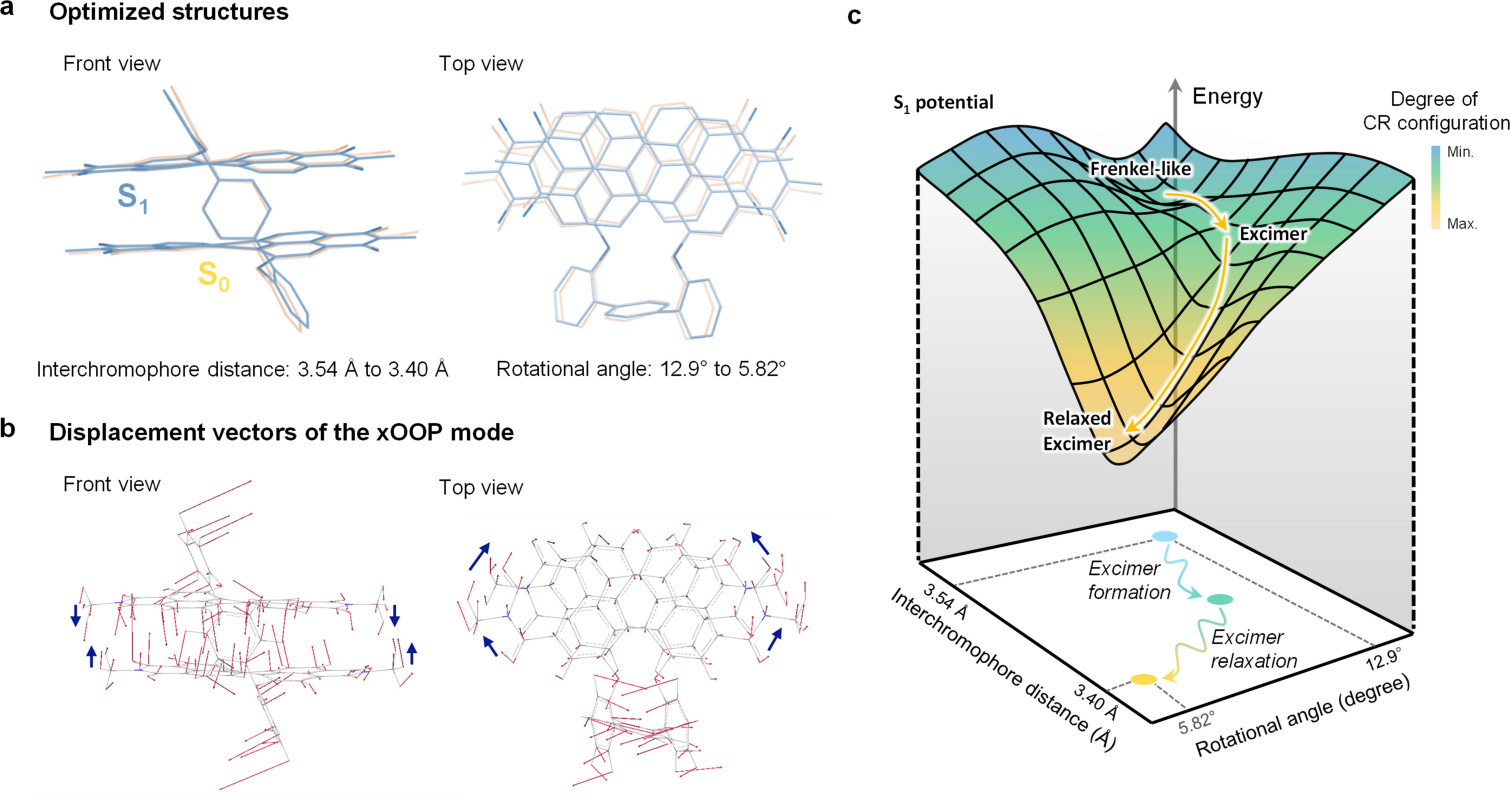
a) Comparison of (TD)‐DFT optimized structures (*ω*B97X‐D/6‐31g(d)) between the S_0_ (yellow) and S_1_ (blue) states. Hydrogens are omitted for clarity. Changes in the interchromophore distance and rotational angle values are included. b) Displacement vectors of the xOOP mode (103.53 cm^−1^, a scaling factor 0.95 is applied). The blue arrows guide the direction of the displacement vectors for clarity. c) Schematic illustration of excimer formation triggered by structural dynamics along the interchromophore coordinate.

To support that structural relaxation is the essential prerequisite for the excimer formation, the temperature‐dependent steady‐state absorption and fluorescence measurements from 77 to 297 K were carried out (Figure 4) in 2‐methyltetrahydrofuran (2‐MeTHF). Interestingly, we observed a sudden spectral jump in the fluorescence spectra near 147 K (the melting point of 2‐MeTHF) (Figure 4b), whereas the absorption spectra maintain similar spectral shapes with minor changes of peak positions and bandwidths (Figure 4a). In completely frozen solution below the meting point of 2‐MeTHF (T<147 K), the Frenkel‐like fluorescence was observed, which is evident from clear vibronic features in the spectra. In sharp contrast, the fluorescence spectra near 147 K dramatically changed into the broad excimer‐like feature with a significant intensity drop. Similar spectral behaviors have been reported before in another PBI dimer molecule but have not been interpreted by relating with temperature‐dependent absorption behaviors in detail.^[56]^ Given that the minor change of temperature‐dependent absorption indicates similar S_0_ structures irrespective of the solvent phase (frozen or melted), the striking change of fluorescence spectra near the melting point supports that the structural rearrangement is an essential prerequisite for the excimer formation. Although we could not perform temperature‐dependent TR‐ISRS measurements due to experimental difficulties, additional TCSPC and TA experiments clearly confirm that the relaxed S_1_ states at 77 and 297 K have different characteristics in terms of their electronic structures (Figure S17).

(TD‐)DFT calculations (*ω*B97X‐D/6‐31g(d) level) show that interchromophore distance of 3.54 Å in the S_0_ geometry becomes closer to 3.40 Å in the S_1_ structure and rotational angles decrease from 12.9° to 5.82° (Figure 5a). Interestingly, these changes entirely coincide with the direction of displacement vectors of the xOOP mode (Figure 5b), highlighting its role as a promotor mode. Although we could not obtain reliable frequency calculation results in the excited state, our TR‐ISRS data showing frequency blue‐shifts upon excimer formation and relaxation can be regarded as vibrational snapshots experimentally reproducing structural evolution estimated through theory, if we consider the generic relation of the distance between two units and frequency values (short distance—high‐frequency relationship). In addition, these structural changes could trigger the perturbation of short‐range CT interaction. The quantum simulation obtained by the restricted active‐space configuration interaction method with double spin‐flip^[57]^ (RAS‐2SF) method shows that the CR configuration in the first optically allowed state (3 %) becomes more significant with evolving into the relaxed excimer geometry (12 %), which is in line with previous calculation results by TD‐DFT.^[44]^

Consequentially, we point out the crucial features of the excimer formation mechanism; Firstly, in numerous co‐facial PBI stacks, including **Bis‐PBI**, similar rate constants of excimer formation (*k*=(≈200 fs)^−1^) were observed irrespective of the external environment^[21]^ (e.g., solvent polarity) and exciton coupling strengths.[^13^, ^18^, ^20^, ^23^, ^29^, ^45^] Secondly, the time constant of the excimer formation corresponds to that of ultrafast structural rearrangement along the interchromophore coordinate. Thirdly, the excited states of **Bis‐PBI** are not pure Frenkel or CT diabats (Tables S3–S4), but rather (quasi)adiabatic states which consist of different degrees of LE and CR configurations.[^10^, ^44^] These features are highly similar to those observed in an adiabatic singlet fission reaction.^[58]^ Especially, in an adiabatic regime, the nuclear rearrangement determines the transition rate. Taken together, our TR‐ISRS results strictly illustrate that the S_1_ state in PBI stacks follows the potential energy surface along the nuclear coordinates and the ultrafast structural rearrangement provokes the adiabatic transformation from the Frenkel‐like to excimer‐like characters within the same S_1_ potential (Figure 5c). In detail, after photoexcitation, the Frenkel‐like S_1_ state is populated near the Franck‐Condon region, showing the well‐defined vibronic emission spectra (Figure 4c).[^18^, ^45^] The subsequent ultrafast structural rearrangement through interchromophore coordinates, i.e., the shortening of interchromophore distance, and the decrease in rotational angle, triggers the perturbation of the short‐range CT interaction in the Frenkel‐like S_1_ state, which eventually leads to the excimer formation. Therefore, it can be explained that the loss of vibronic features observed in the transient fluorescence spectra in co‐facially stacked PBIs that we previously reported[^18^, ^45^] originates from the structural relaxation due to the enhancement of CR configuration in the excimer formation. Given the information on the structural change, we suggest an important structural factor for designing OPV materials. In light of the excimer formation, the shortening of interchromophore distance and the decrease in rotational displacement are key reaction coordinates, which is supported by quantum calculations.[^14^, ^15^, ^16^] Thus, the structural restriction along the stacking coordinate is required to hinder the excimer formation in OPV devices.

## Conclusion

In conclusion, we have presented that ultrafast structural changes through the interchromophore vibrational coordinate can trigger the excimer formation by analyzing vibrational snapshots and their time‐dependent behaviors through ultrafast time‐domain Raman spectroscopy. This work demonstrated the vibronic coupling mechanism of the excimer formation of an archetypical PBI dimer stack in light of structural dynamics, which was previously suggested by theoretical simulations. Our observations not only help the researchers in the field clarify the fundamental information on the structural dynamics of the excimer state but also present the guidelines for designing the π‐conjugated organic chromophores of the PBI family^[59]^ and beyond for application in OPV devices.

## Conflict of interest

The authors declare no conflict of interest.

1

## Supporting information

As a service to our authors and readers, this journal provides supporting information supplied by the authors. Such materials are peer reviewed and may be re‐organized for online delivery, but are not copy‐edited or typeset. Technical support issues arising from supporting information (other than missing files) should be addressed to the authors.

Supporting Information

## Data Availability

Research data are not shared.
