# Supplementary material for: Real‐time Observation of Structural Dynamics Triggering Excimer Formation in a Perylene Bisimide Folda‐dimer by Ultrafast Time‐Domain Raman Spectroscopy
Source: Angew Chem Int Ed Engl. 2022 Feb 7;61(13):e202114474. doi: 10.1002/anie.202114474 (PMC9306572; doi:10.1002/anie.202114474)
Supplement: Supplementary file 1 — Supporting Information [file ANIE-61-0-s001.pdf]

Angewandte Chemie
Eine Zeitschrift der Gesellschaft Deutscher Chemiker

Supporting Information

Real-time Observation of Structural Dynamics Triggering Excimer Formation in a Perylene Bisimide Folda-dimer by Ultrafast Time-Domain Raman Spectroscopy

Y. Hong, W. Kim, T. Kim, C. Kaufmann, H. Kim, F. Würthner*, D. Kim**

Supporting Information
©Wiley-VCH 2021
69451 Weinheim, Germany

Real-time Observation of Structural Dynamics Triggering Excimer Formation in a Perylene Bisimide Folda-dimer by Ultrafast Time-domain Raman Spectroscopy

Yongseok Hong[†], Woojae Kim[†], Taeyeon Kim, Christina Kaufmann, Hyungjun Kim[*], Frank Würthner[*], and Dongho Kim[*]

Table of Contents

1. Experimental Details – 2

2. Supporting Figures and Tables – 5

3. References– 19

1. Experimental Details

Sample preparation, steady-state measurements, and quantum calculation.

ref-PBI and **Bis-PBI** were prepared according to the reported methods.[1] Steady-state absorption spectra were measured on a UV/Vis/NIR spectrometer (Varian, Cary5000) and fluorescence spectra were measured on a fluorescence spectrophotometer (Hitachi, F-7000). Fluorescence spectra are spectrally corrected by using correction factor of the fluorescence spectrophotometer. HPLC-grade solvents were purchased from Sigma-Aldrich and used without further purification. Sample concentrations for steady-state absorption and fluorescence measurements of all samples were ~2.5 x 10^{-6} and 1.0 x 10^{-6} M, respectively. According to the previous literature,[1] **ref-PBI** and **Bis-PBI** do not show intermolecular interactions in the concentration range of 10^{-6} – 10^{-3} M region (in trichloroethylene but we confirmed that both in toluene (TOL) and tetrahydrofuran (THF) they do not form large intermolecular aggregates), which means that we can rule out the existence of long-range aggregates for both samples in typical ultrafast optical spectroscopic experiments. For temperature-dependent measurement, a temperature-controlled liquid nitrogen cryostat (Oxford Instruments, Optistat DN) was used. And, we used 2-methyltetrahydrofuran (2-MeTHF) instead of THF. Temperatures were maintained to within ±0.05 K and allowed to equilibrate for 30 min before measurement. Quantum mechanical calculations were carried out with Gaussian 16 program suite.[2] All calculations (DFT) were performed based on *ω*B97X-D employing a basis set consisting of 6-31g(d) for all atoms.[3]. For all calculations, alkyl groups substituted on imide positions were replaced by methyl groups to solely investigate the effect of intermolecular interaction of PBIs. The geometry optimizations in the ground and excited state are based on previous reports.[1,4] We found that there is no big difference between *ω*B97X-D (this work) and CAM-B3LYP-D3 (ref. 4) functional. Details for RAS-2SF calculations are described in the page 16 in this Supporting Information.

Femtosecond transient absorption spectroscopy with a Ti:Sapphire amplifier

The transient absorption (TA) spectroscopy setup has been described in detail elsewhere.[10-12] In brief, a Ti:sapphire regenerative amplifier (Integra-C, Quantronix, 800 nm, 1 mJ, 1 kHz, 100 fs,) was used as a fundamental laser source of femtosecond transient absorption spectrometer. White light continuum (WLC) probe pulses were generated using CaF2 window (3mm thick, for visible region) and YAG window (4 mm thick, for NIR region) by focusing a small portion of the transmitted fundamental pulses. Pump pulses (510 nm) were generated through a commercial collinear optical parametric amplifier (Palitra, Quantronix). The pulse energy of the pump was attenuated to 300 nJ and its polarization was set at the magic angle to the vertically polarized probe by using a half-wave plate (Thorlabs) and a Glan-laser polarizer (Thorlabs). A 2 mm path length quartz cell (21/Q/2, Starna) was used and the optical density (OD) of the sample was about 0.5 (c_0 = 6.3 x 10^{-5} M). TA spectra were measured in a shot-to-shot fashion by modulating pump pulses at 500 Hz using an optical chopper (MC1F10, Thorlabs). With the optical Kerr effect measurements by *n*-hexane, cross-correlation FWHM (full-width at half-maximum) in the TA experiments was estimated to be about 200-300 fs depending on the probe wavelength and the chirp of WLC probe pulses was measured to be 1.2 ps in the 450-1300 nm region.

Time-resolved impulsive stimulated Raman spectroscopy (TR-ISRS)

A schematic diagram of the TR-ISRS is shown in Supplementary Figure S1 and the details were described in elsewhere.[13,14] Briefly, a Yb:KGW regenerative amplifier (PHAROS-SP-1.5mJ, Light Conversion, 1030 nm, 600 μJ, 10 kHz, 176 fs) was used as the main source for TR-ISRS. Actinic pump (P_1, 510 nm, ~ 170 fs) is generated by a commercial collinear optical parametric amplifier (ORPHEUS, Light Conversion) combined with a second-harmonic generation stage (LYRA-SH, Light Conversion). A home-built noncollinear optical parametric amplifier (NOPA) generates broadband pulses covering the near-infrared region (700-900 nm, compressed to sub-10 fs by chirped mirrors and wedges and characterized by SHG-FROG) and they were used as Raman pump (P_2) and probe (P_3) pulses after dividing by a beam splitter (Venteon). At the sample position, the energies (and $1/e^2$ beam diameters) of the P_1, P_2, and P_3 pulses were 200 nJ (120 μm), 120 nJ (65 μm), and 3 nJ (55 μm), respectively, and all pulses were horizontally polarized. A 500 μm optical path length flow cell with ultrathin wall apertures (48/UTWA2/Q/0.5, Starna) was used and the 2.5 ml sample solution (OD for a 500 μm cell = 0.8 at absorption maximum, c_0 = 5.0 x 10^{-4} M) was flowed by a microannular gear pump (mzr-4622 M2.1, HNP Mikrosysteme). The P_3 and the reference pulses were detected using the Si photodiodes (S2281-04, Hamamatsu) without any filters for open-band detection to minimize the contribution of vibrational coherences from the ground-state and solvent molecules. The P_2 pulse is modulated at 5 kHz by a mechanical chopper (MC1F60, Thorlabs), which allows data processing in a shot-to-shot fashion. Especially, for TA experiments with P_1 and P_3 pulses (data in Figures 1d and 1e), The P_2 pulse is blocked by a beam block and the P_1 pulse is modulated at 5 kHz by a mechenical chopper (MC1F60, Thorlabs) to get P_1-induced TA signals.

Sub-10-fs transient absorption spectroscopy with a Yb:KGW amplifier

A schematic diagram of the sub-10-fs TA is shown in Supplementary Figure S1. For this setup, we use the same Yb:KGW regenerative amplifier (PHAROS-SP-1.5mJ, Light Conversion, 1030 nm, 600 μJ, 10 kHz, 176 fs). Visible NOPA (Vis-NOPA) pulse is generated at BBO by overlapping 343 nm third-harmonic generation (THG) pump and pre-compressed seed pulse (white light continuum) generated

by using a 3 mm sapphire plate. After amplification, Vis-NOPA is compressed via chirped mirrors, followed by fine-tuning with a wedge prism pair. The temporal width reaches near 7 fs characterized by SHG-FROG. The probe pulse is generated with a 2 mm YAG plate, allowing us to detect the 600-900 nm region. The time delay between pump and probe is controlled by the motorized linear stage (M-VP-25XA (Newport)). Polarizations of two pulses are parallel at the sample position to maximize vibrational coherence signals. The signals are obtained by a CCD detector (Stressing, FL-3030). As in the TR-ISRS experiment, in order to minimize GDD, we use an ultrathin wall aperture (500 μm UVFS) flow cell (48/UTWA2/Q/0.2, Starna) with 200 μm optical path length. Also, a microannular gear pump (mzr-4622 M2.1) with Teflon tubing is used for flowing the sample solutions which can remove the thermal-lens effect due to the 10 kHz repetition rate of the laser and avoid photo-degradation of the sample. We usually prepare 2–3 ml sample solutions for the experiments (c_0 = 5.0 x 10^{-4} M). The pump pulse is modulated at 5 kHz by a mechanical chopper (MC1F60, Thorlabs), which allows data processing in a shot-to-shot fashion. 30000 x 2 pulses are averaged for each time delay.

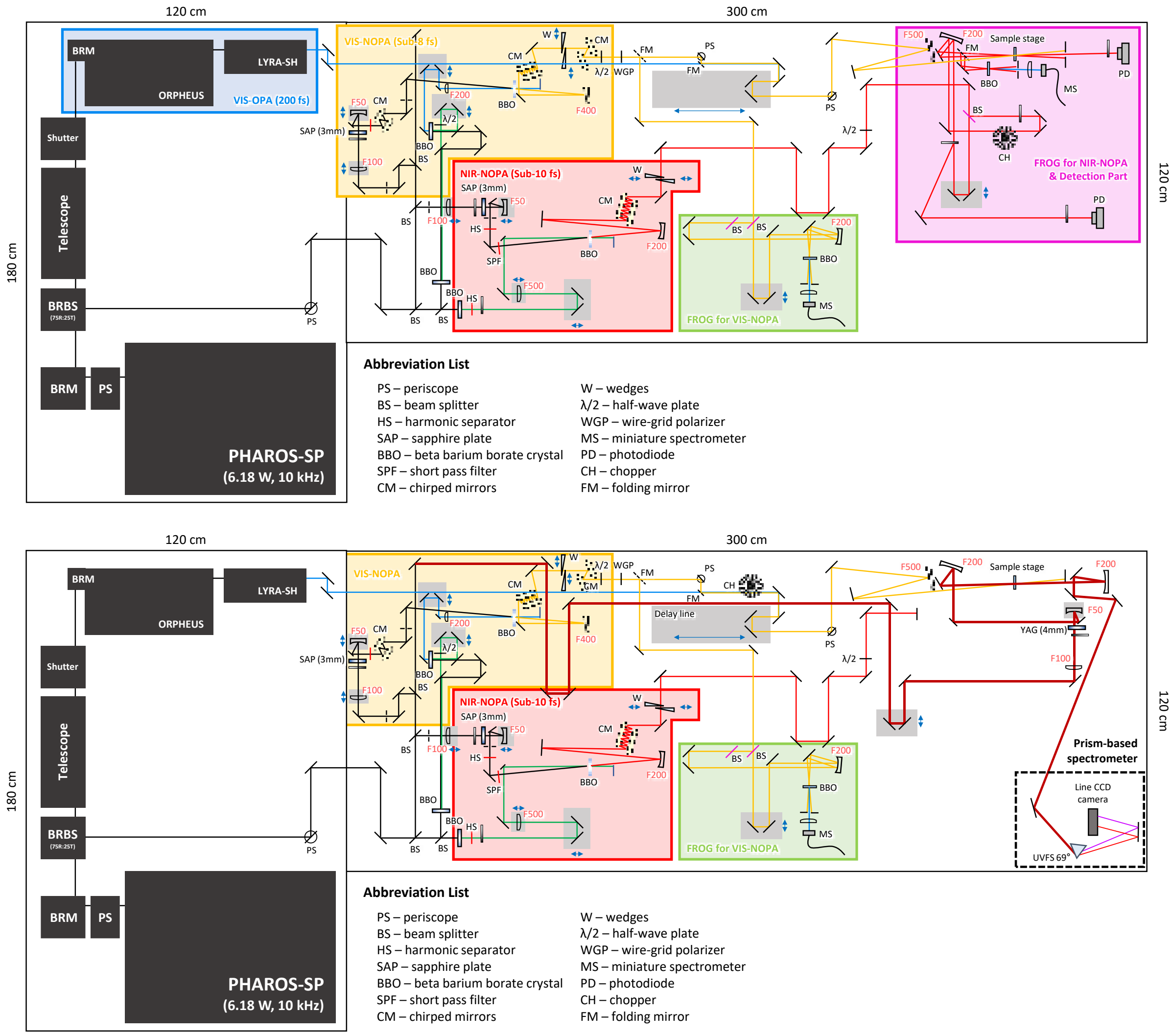

Figure S1. TR-ISRS and ultrafast TA setup.
Schematic diagram of TR-ISRS setup using OPA as the actinic pump (P_1) and NIR-NOPA (P_2 and P_3) as the Raman pump and probe (top). Schematic diagram of transient absorption setup using VIS- or NIR-NOPA as the pump and white light continuum as the probe. (bottom)

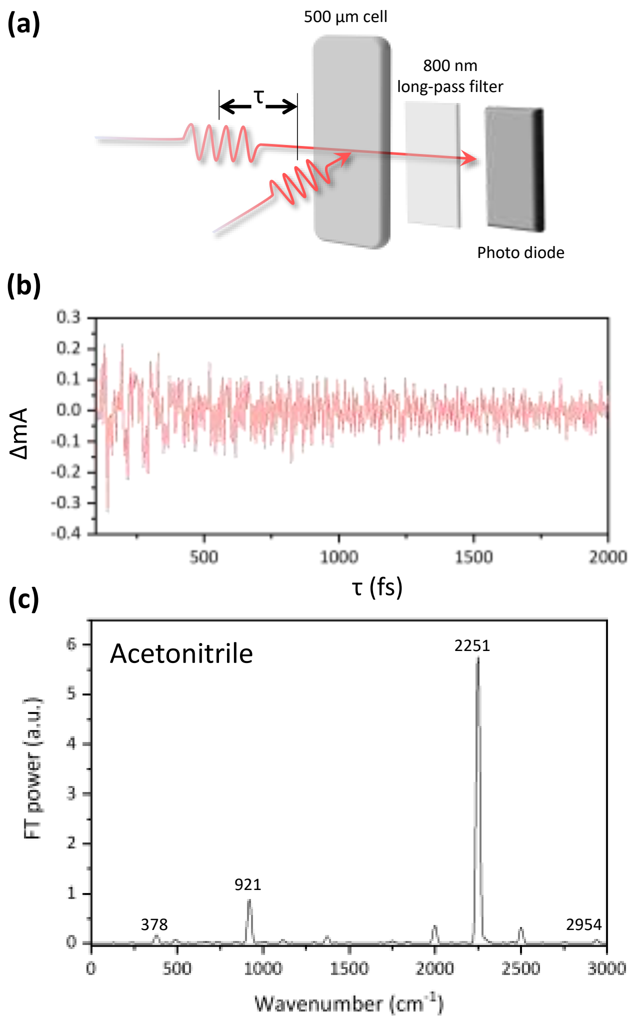

Figure S2. Non-resonant ISRS measurements for neat acetonitrile. (a) Experimental scheme of non-resonant ISRS measurements. (b) Raw non-resonant ISRS signals of acetonitrile. (c) Fourier transform power spectrum of acetonitrile obtained after windowing (Hanning function) and zero-padding for oscillatory residual. Fourier transform power spectrum acetonitrile reveals a vibrational peak of 2954 cm^{-1} (C-H stretch).

2. Supporting Figures & Tables

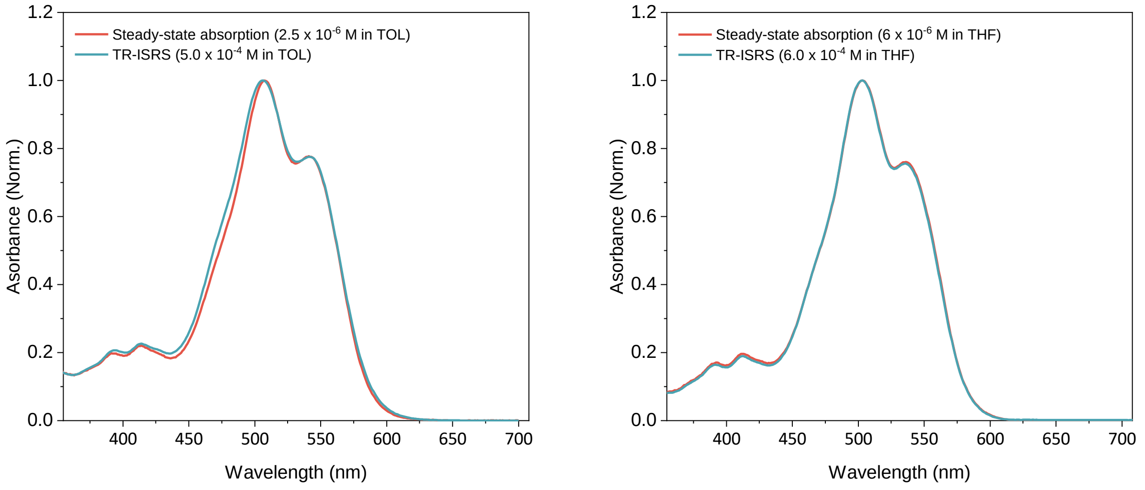

Figure S3. The concentration-dependent steady-state absorption spectra of **Bis-PBI** in TOL (left) and THF (right). The red/blue lines indicate the dilute/concentrated conditions for steady-state/TR-ISRS measurements.

Table S1. The optical properties of **Bis-PBI** in the various solvents.

**Bis-PBI**	QY(%)	τ^a (ns)	$k_{rad}(10^7)$	$k_{nonrad}(10^7)$
MCH	13.1	17.3	0.76	5.03
TOL	12	17.1	0.71	5.18
2me-THF (RT)	7.6	14.9	0.51	6.2
2me-THF (77K)	12.7	16.4	0.77	5.33
BCN	1.1	6.1	0.19	16.2

*The relative quantum yields are measured by rhodamine-6G in ethanol as a reference.
[a] The fluorescence lifetime is calculated by averaging the multi-exponential decay profiles.

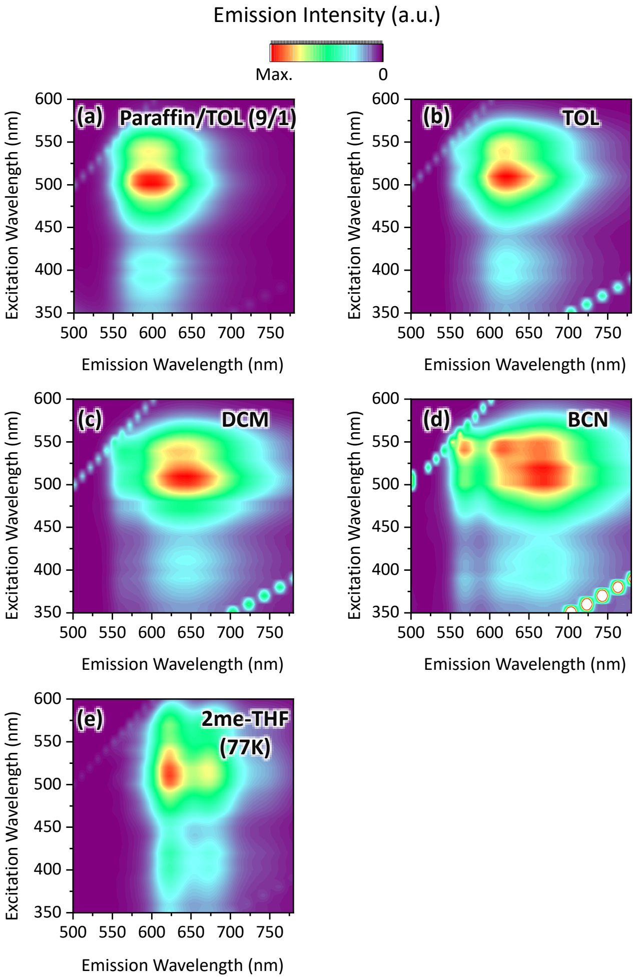

Figure S4. The two-dimensional emission-excitation map for **Bis-PBI** in (a) Paraffin/TOL (9/1), (b) TOL, (c) DCM, (d) BCN, and (e) 2-MeTHF (77K)

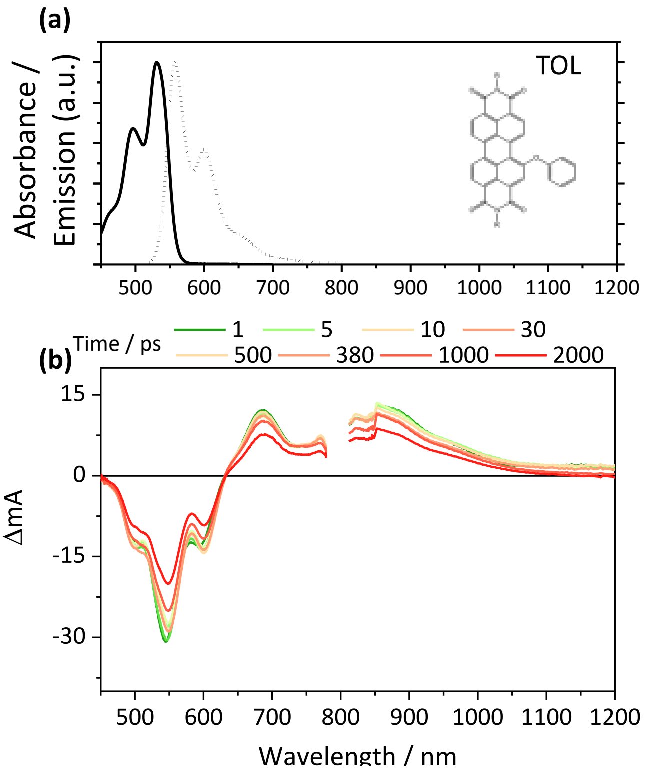

Figure S5. (a) Steady-state absorption and emission spectra of **ref-PBI** in TOL. (b) The representative TA spectra of **ref-PBI** at different delay times.

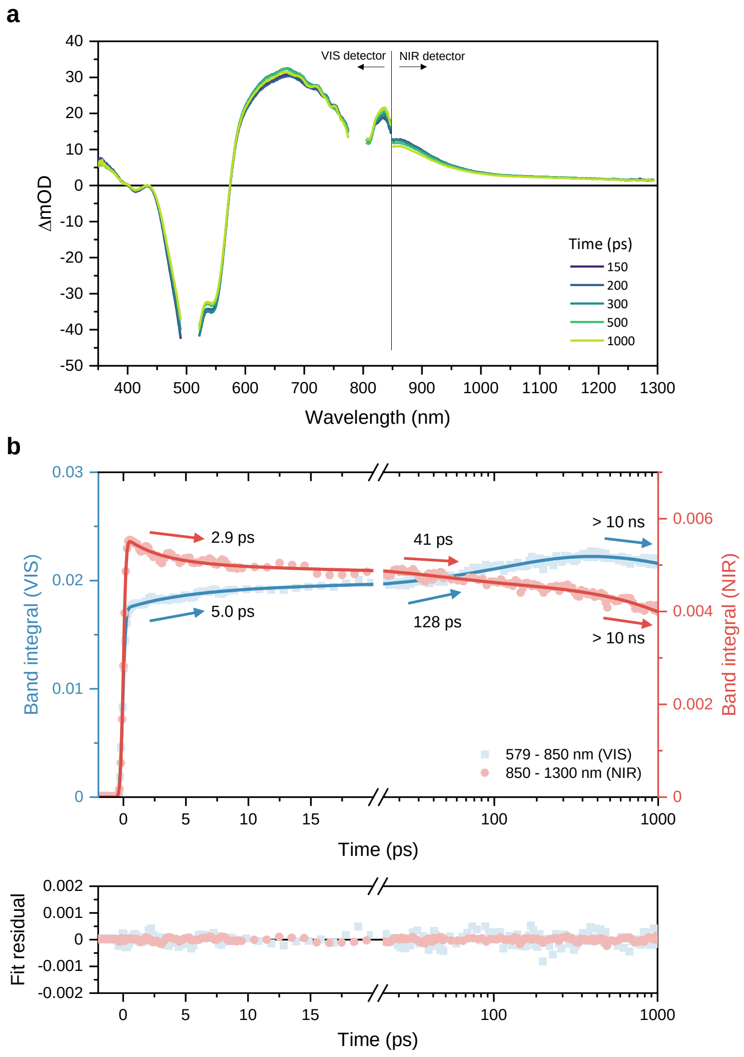

Figure S6. (a) The representative transient absorption spectra of **Bis-PBI** in TOL. (b) Band integral profiles (top) and fit residuals (bottom).

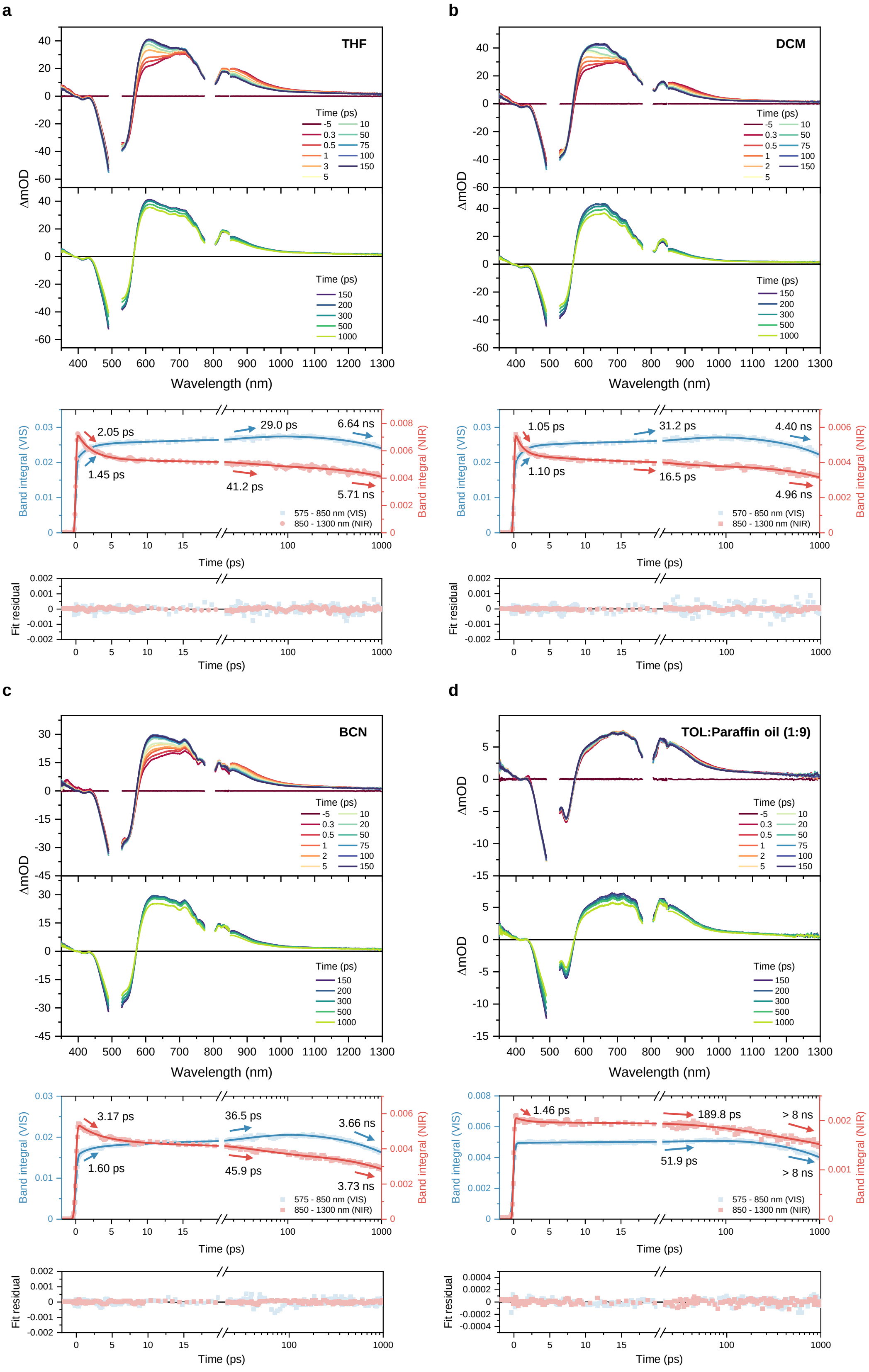

Figure S7. (a-d) Solvent dependent transient absorption spectra and band integral kinetics of **Bis-PBI** in THF (a), DCM (b), BCN (c), TOL:Paraffin oil (1:9) (d). The representative spectra (top), band integral (middle), and fit residuals (bottom).

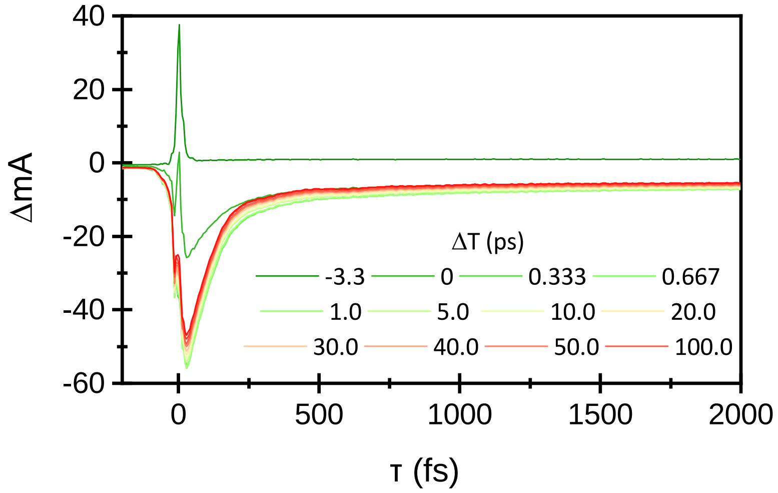

Figure S8. Raw TR-ISRS kinetic profiles of **Bis-PBI** in TOL.

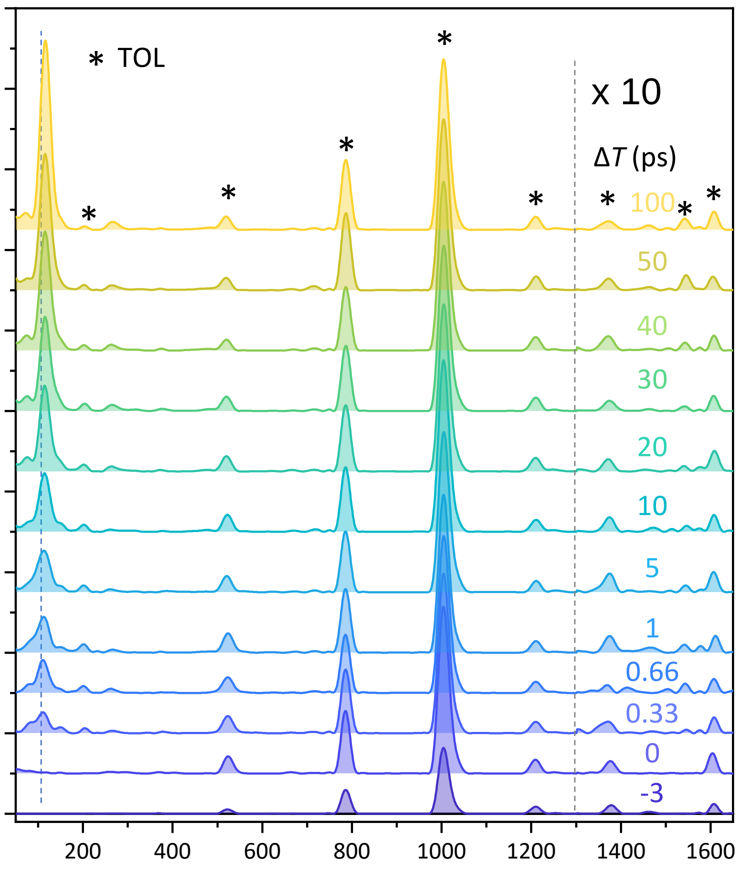

Figure S9. Time-resolved Raman spectra of **Bis-PBI** in TOL from low- to high-frequency range. Asterisks indicate solvent Raman bands. The frequency region above 1300 cm-1 is multiplied by 10 times to clarify the high-frequency region.

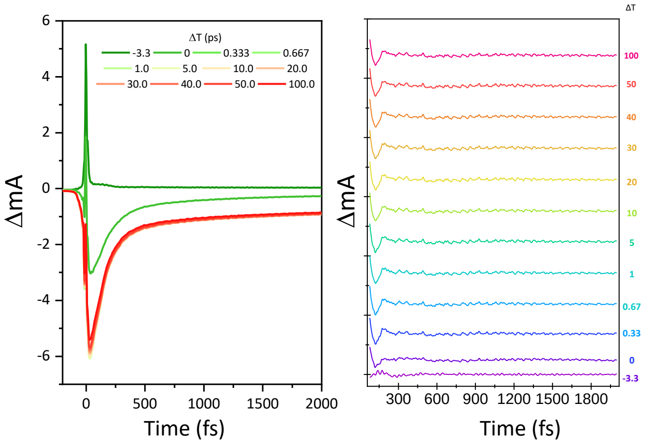

Figure S10. Raw TR-ISRS kinetic profiles and oscillatory residuals extracted from multiexponential fits to the kinetic traces at each T for **ref-PBI** in TOL.

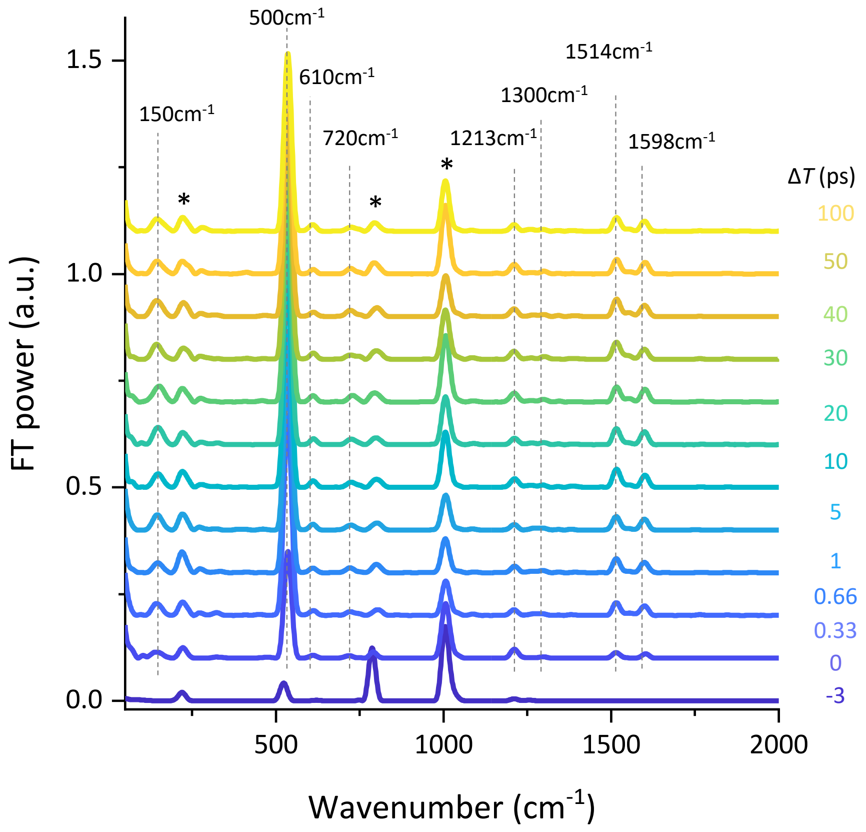

Figure S11. Time-resolved Raman spectra of **ref-PBI** in TOL. Asterisks indicate solvent Raman bands.

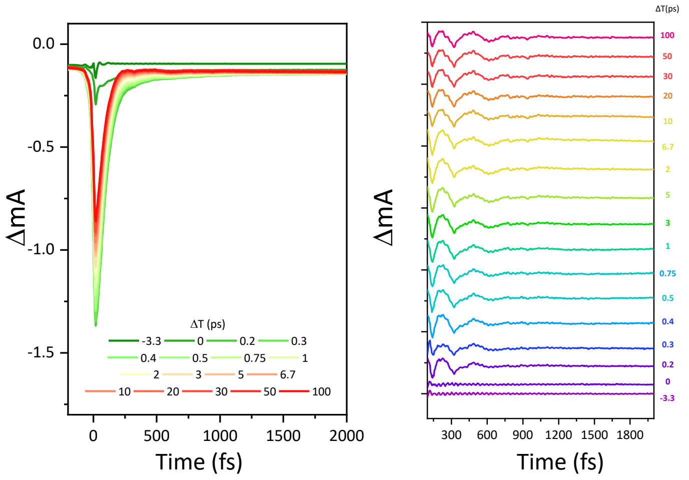

Figure S12. Raw TR-ISRS kinetic profiles and oscillatory residuals extracted from multiexponential fits to the kinetic traces at each T for **Bis-PBI** in THF.

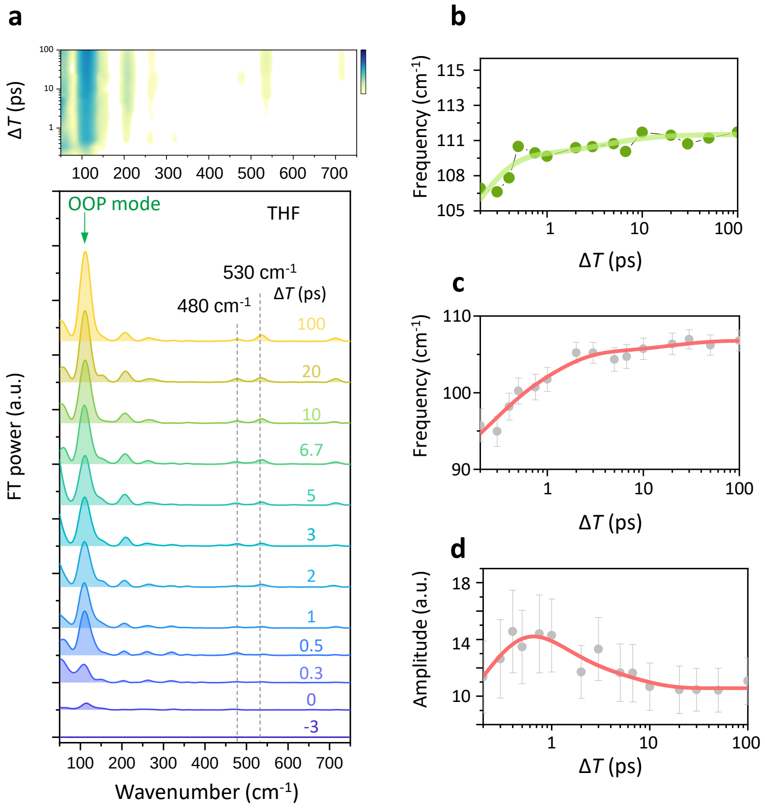

Figure S13. Time-resolved Raman spectra of **Bis-PBI** in THF. (a) The representative excited-state Raman spectra. The arrow indicates the xOOP mode. (b) the temporal trace for the frequency of xOOP mode obtained by gaussian fits in frequency-domain. (c) the temporal trace for the frequency of xOOP mode obtained by the sinusoidal fitting in time-domain. (d) the kinetics for oscillatory amplitude of xOOP mode obtained by the sinusoidal fitting in time-domain.

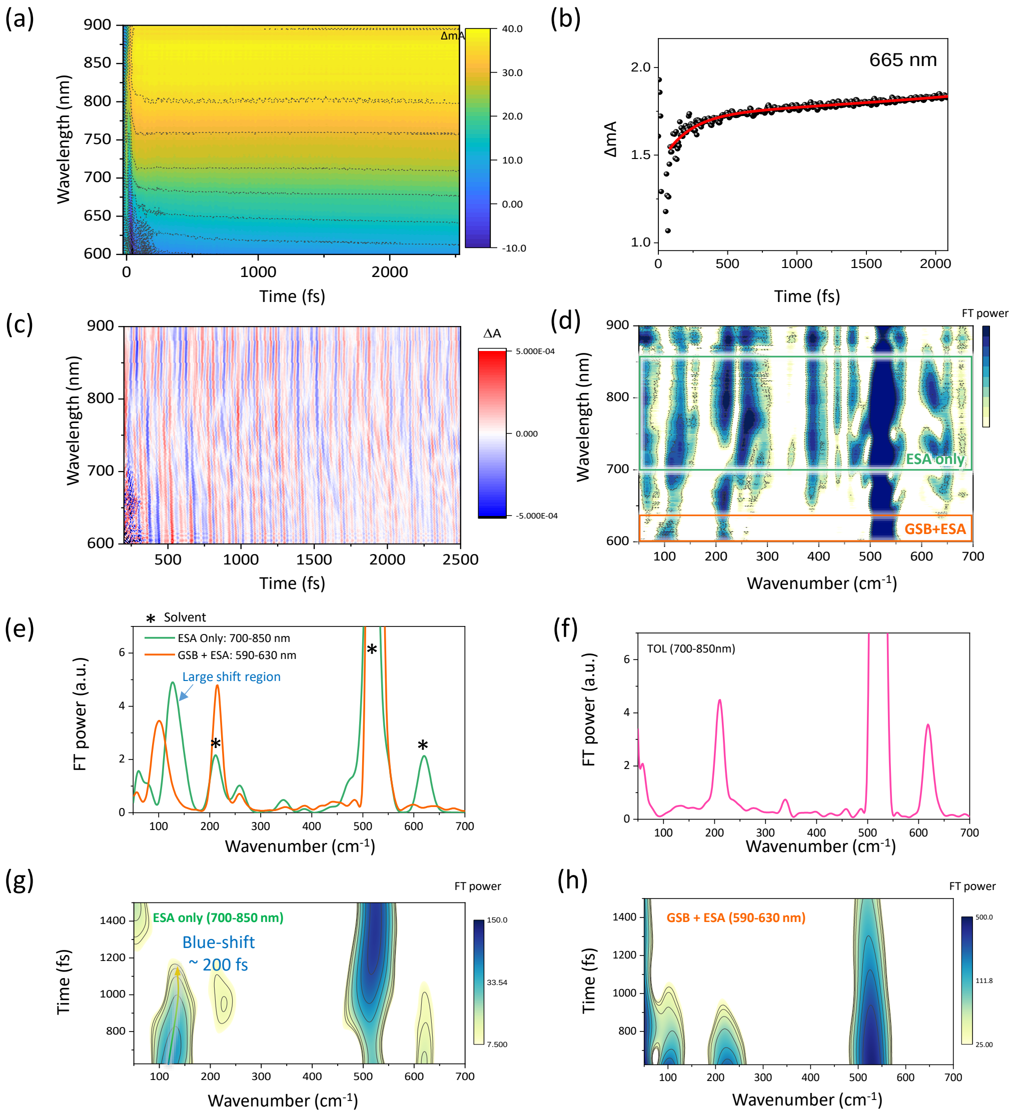

Figure S14. TA results of **Bis-PBI** in TOL using sub-10fs Vis-NOPA. (a) two-dimensional (2D) contour map. (b) Kinetics at the probe wavelength of 665 nm. (c) 2D contour map of oscillatory residuals. (d) 2D FT power map. The green and red dashed areas correspond to the ESA (700-850 nm) and GSB+ESA (580-630 nm) regions in Figure S13e. (e) The averaged FT power spectra between 700-850 nm (yellow line) and 590-630 nm (green line). (f) The averaged FT power spectra of TOL between 700-850 nm. The asterisk indicates the signal of TOL (g) Sliding window Fourier transform result using Hanning window function (FWHM=1.1 ps) at the averaged region between 700-850 nm. (h) Sliding window Fourier transform result using Hanning window function (FWHM=1.1 ps) at the averaged region between 590-630 nm.

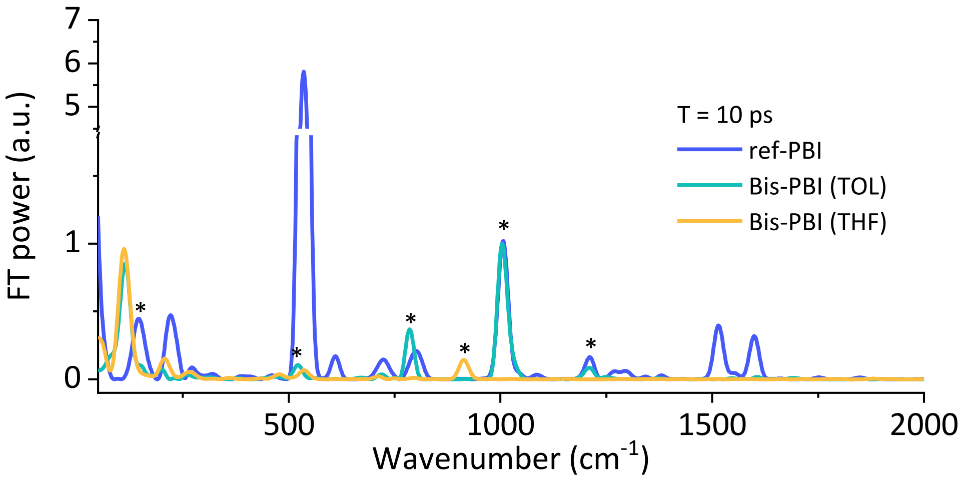

Figure S15. The comparison of the excited state Raman spectra of r**ef-PBI** (blue), **Bis-PBI** in TOL (green), and **Bis-PBI** in THF (yellow) at the time interval of T=10 ps. Asterisks indicate solvent Raman bands.

Table S2. The additional parameters for sinusoidal fitting of the VCs of Bis-PBI in TOL

	A_2	ν_2	τ_2	Φ_2
2nd parameter	0.02264	160 cm-1	41 fs	0.22 π

Discussion on analysis of the excited-state Raman

Here, we would like to emphasize a few findings to rationalize our analysis. 1) Analysis of the transient absorption: Although the isosbestic point is around 800 nm, the broadband TA spectra indicated that the ESA band over the NIR NOPA region holds its spectral feature with time (Figure S15). This suggests that the electronic structures in the Frenkel-like state could be quite similar to those in the excimer state. Furthermore, the initial TA spectrum in a polar solvent is different from those in a weak polar solvent (Figure 2 and S4-S5), indicating that the initial Frenkel-like state is also mixed with the CT state like the excimer state. 2) Temperature-dependent experiments: The temperature-dependent experiments strongly support that the excimer formation process is accompanied by structural changes. It is noted that time-resolved fluorescence results demonstrated the changes in the emission spectra with the excimer formation process (i.e., the inversion of $A_{0\text{-}0}/A_{0\text{-}1}$) in the previous report[4]. While the Frenkel-like emission was observed below the melting point, the emission spectrum dramatically changed into an excimer-like feature above the melting point. The striking change in the emission spectrum above the melting point is a strong evidence for the structural dynamics in the excimer formation. 3) Excimer formation observed in various PBI dimers: We recall the excimer formation process in numerous PBI dimers that show different coupling strengths as described in the main text. The excimer formation process in PBI dimers occurs with the ultrafast timescale of 200 fs irrespective of their coupling strength. Furthermore, as we mentioned before, negligible changes in TA spectra were observed during the excimer formation process. Overall, these results suggest that the excimer formation process is adiabatic rather than diabatic. 4) Gradual shifts of xOOP mode: If the transition is close to the diabatic process, the isosbestic point is expected. However, Raman dispersions of xOOP mode show gradual blue-shifts without the isosbestic point. Especially, the short-time Fourier transform (STFT) using broadband TA also shows a gradual blue-shift in Raman frequency around 110 cm^{-1} (Figure S13). The broad visible NOPA (< 10 fs) triggers the excited state wavepackets near the FC geometry. Furthermore, if xOOP mode is an exclusive feature of the excimer state, the distinct rise (OOP mode for excimer state) and decay (initial OOP mode for Frenkel-like state) should be observed. However, STFT only shows gradual blue-shifts in Raman frequency of xOOP mode. The STFT result reveals that the xOOP mode is not the exclusive signature of the excimer state itself. Taken all together, the excimer formation process is not only a change of the configuration percentage in admixture of locally excited (LE) and charge transfer (CT) wavefunctions but also accompanied by the structural change.

Also, we present the averaging procedure to improve the quality of experimental results such as the frequency and power of the excited state Raman spectra. As we described in the experimental details, we used zero-padding and Hanning window function to improve the quality of FT power spectra. To minimize the artifact induced by the long-term effects such as the fluctuation of the excitation power and/or laser stability, the set of all time delays (ΔT) was measured repeatedly; (set 1 [-3.3 → 0 → · · · → 100 ps] → set 2 [-3.3 → 0 → · · · → 100 ps] → ···). Especially, Figure S16 indicate that FT power spectra are converged after 15 or more of raw TR-ISRS decay averages. Furthermore, other noises were significantly suppressed by the averaging effect. As shown in Figure S16, the comparison of the excited-state Raman spectra at 0.33 and 1 ps depending on the averaged decays confirm the following points: 1) the frequency of xOOP mode is blue-shifted and 2) the amplitude of xOOP mode increases.

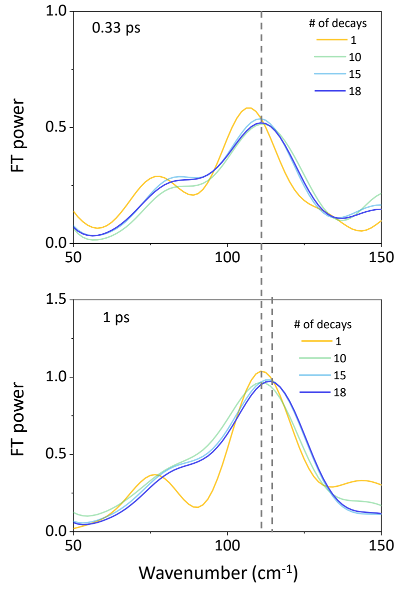

Figure S16. The averaging effects. The excited-state Raman spectrum of **Bis-PBI** at the ΔT = 0.33 (top) and 1 (bottom) ps. The dashed lines show the center position of the Raman frequency.

Additional discussion on temperature-dependent TA and time-resolved fluorescence measurements

The time-resolved fluorescence spectra at 77 K and 297 K indicate nearly similar decay profiles and the absence of dramatic spectral evolution in the overall spectral range (Figures S20a and S20b), suggesting the lack of conformational heterogeneity. In contrast, the initial TA spectral features in frozen solution show different ESA structures compared to those in solution at room temperature (Figures S20c and S20d). The ESA band around 600 nm at 77 K decays with the time constant of 2 ps, while the biexponential rise of the ESA region at 297 K (4 and 16 ps) was observed. The initial state relaxes to the bright state with a time constant of 2 ps in the frozen solution, while the excimer state at the temperature above the melting point was formed by the ultrafast structural rearrangement.

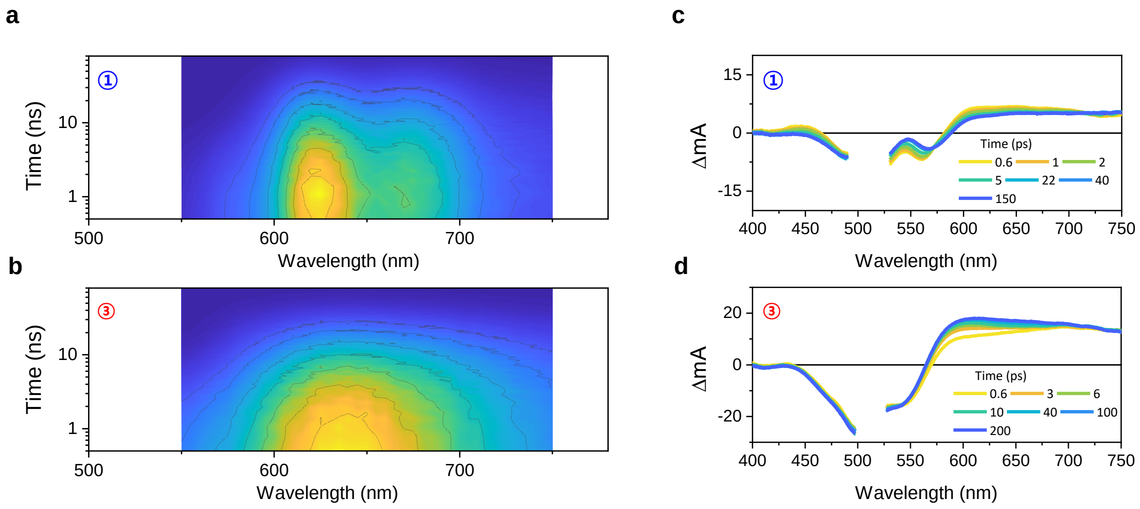

Figure S17. Time-resolved fluorescence spectra at (a) 77 K, (b) 297 K and TA spectra at specific delay times at (c) 77 K and (d) 297 K.

RAS-2SF calculation

We performed quantum chemical simulations to characterize electronic states associated with the excimer. The ground state (S_0) geometry obtained by DFT (ωB97X-D/6-31G(d)) was provided in the previous paper. The first excited singlet state (S_1) geometry was optimized with time-dependent DFT with the same functionals and basis sets used for the ground state one. We would like to provide more detailed explanations on the selection of ωB97X-D for this work. It is well known that the charge transfer transition energy is underestimated by TD-DFT, which is due to the wrong asymptotic behavior of Coulomb potential. While generalized gradient approximation functionals and even hybrid functionals suffer from this problem, range-separated hybrid functionals can improve the description of asymptotic behavior of Coulomb potential by separating short- and long-range exchange interactions. The contribution of Hartree-Fock exchange increases with the interelectronic distance, and becomes 100 % at infinity. This approach recovers the correct asymptotic behavior of Coulomb potential, and can provide significant improvements for charge transfer energies. In this work, we employed one of range-separated hybrid functional, ωB97X-D, to optimize S_0 and S_1 structures. Also, ωB97X-D functional proves itself to be capable of providing quantitative understanding in the electronic structures of excited states of PBI molecules.[13,14] Therefore, we can expect that the S_0 and S_1 geometries of Bis-PBI optimized by ωB97X-D/6-31G(d) are quite reasonable, and can provide reliable descriptions of electronic structures.

Restricted active-space configuration interaction method with double spin-flip (RAS-2SF) calculations were performed to characterize the excimer-relevant electronic states in terms of diabatic states such as a ground state (GS), a local excitation (LE), a multiexciton state (ME) and charge resonance (CR).[15] We used the quintet reference state with four electrons in four active orbitals to include ME diabat which can play a role in stacked PBI structures. Even though RAS-2SF can achieve the balanced description of singly and doubly excited states, the lack of electron dynamic correlation results in overestimation of transition energies. Electron dynamic correlation can be partly recovered by the comparison with DFT energies.[13] TDDFT, constrained DFT, and unrestricted high-spin DFT computations were used to obtain local exciton, charge-resonance, and quintet state energies, respectively. The energies of adiabatic states from RAS-2SF with dominant local exciton, charge-resonance, and TT multiexciton diabatic character were adjusted to reproduce corresponding DFT energies. For DFT energies, we used ωB97X-D/6-31G(d). Table S3 and Table S4 list the corrected RAS-2SF energies at the ground state (S_0) and the first singlet excited state (S_1) geometries, respectively.

All quantum chemical simulations were performed with Q-Chem 5.1.[16]

Table S3. Corrected RAS-2SF excitation energies (in eV) and adiabatic wavefunction composition (%) of excimer-relevant states at the ground state (S_0) geometry.

%	Energy	f (oscillator strength)	GS	LE	ME	CR
S_1	2.06	0.039	24%	35%	29%	12%
S_2	2.49	0.004	1%	8%	18%	73%
S_3	2.60	0.115	0%	3%	1%	96%
**S_4 (optically allowed state)**	**2.66**	**2.840**	**0%**	**83%**	**14%**	**3%**
S_5	2.85	0.396	0%	71%	26%	3%
S_6	3.44	0.003	0%	4%	84%	12%

Table S4. Corrected RAS-2SF excitation energies (in eV) and adiabatic wavefunction composition (%) of excimer-relevant state at the first singlet excited state (S_1) geometry.

%	Energy	f (oscillator strength)	GS	LE	ME	CR
S_1	1.98	0.978	0%	86%	2%	12%
S_2	2.10	0.032	2%	0%	0%	98%

References

[1] Kaufmann, C.; Bialas, D.; Stolte, M.; Würthner, F. *J. Am. Chem. Soc.* **2018**, *140* (31), 9986–9995.

[2] Gaussian 16, Revision C.01, Frisch, M. J.; Trucks, G. W.; Schlegel, H. B.; Scuseria, G. E.; Robb, M. A.; Cheeseman, J. R.; Scalmani, G.; Barone, V.; Petersson, G. A.; Nakatsuji, H.; Li, X.; Caricato, M.; Marenich, A. V.; Bloino, J.; Janesko, B. G.; Gomperts, R.; Mennucci, B.; Hratchian, H. P.; Ortiz, J. V.; Izmaylov, A. F.; Sonnenberg, J. L.; Williams-Young, D.; Ding, F.; Lipparini, F.; Egidi, F.; Goings, J.; Peng, B.; Petrone, A.; Henderson, T.; Ranasinghe, D.; Zakrzewski, V. G.; Gao, J.; Rega, N.; Zheng, G.; Liang, W.; Hada, M.; Ehara, M.; Toyota, K.; Fukuda, R.; Hasegawa, J.; Ishida, M.; Nakajima, T.; Honda, Y.; Kitao, O.; Nakai, H.; Vreven, T.; Throssell, K.; Montgomery, J. A., Jr.; Peralta, J. E.; Ogliaro, F.; Bearpark, M. J.; Heyd, J. J.; Brothers, E. N.; Kudin, K. N.; Staroverov, V. N.; Keith, T. A.; Kobayashi, R.; Normand, J.; Raghavachari, K.; Rendell, A. P.; Burant, J. C.; Iyengar, S. S.; Tomasi, J.; Cossi, M.; Millam, J. M.; Klene, M.; Adamo, C.; Cammi, R.; Ochterski, J. W.; Martin, R. L.; Morokuma, K.; Farkas, O.; Foresman, J. B.; Fox, D. J. **2016.** Gaussian, Inc., Wallingford CT,.

[3] Yanai, T., Tew, D. P. & Handy, N. C. *Chem. Phys. Lett.* **2004**, *393*, 51–57.

[4] Hong, Y.; Kim, J.; Kim, W.; Kaufmann, C.; Kim, H.; Würthner, F.; Kim, D. *J. Am. Chem. Soc.* **2020**, *142* (17), 7845–7857.

[5] Kuramochi, H.; Takeuchi, S.; Tahara, T. *Rev. Sci. Instrum.* **2016**, *87*, 043103.

[6] Liebel, M.; Schnedermann, C.; Kukura, P. *Opt. Lett.* **2014**, *39* (14), 4112.

[7] Grupp, A.; Budweg, A.; Fischer, M. P.; Allerbeck, J.; Soavi, G.; Leitenstorfer, A.; Brida, D. *J. Opt. (United Kingdom)* **2018**, *20*, 104005.

[8] Teo, S. M.; Ofori-Okai, B. K.; Werley, C. A.; Nelson, K. A. *Rev. Sci. Instrum.* **2015**, *86,* 051301.

[9] Trebino, R.; Delong, K. W.; Fittinghoff, D. N.; Sweetser, J. N.; Richman, B. A.; Krumbu, M. A.; Kane, D. J. **1997**, *68* (9), 3277–3295.

[10] Kim, T.; Kim, W.; Vakuliuk, O.; Gryko, D. T.; Kim, D. *J. Am. Chem. Soc.* **2020**, *142* (3), 1564–1573.

[11] Kim, W.; Kim, T.; Kang, S.; Hong, Y.; Würthner, F.; Kim, D. *Angew.. Chem. Int. Ed.* **2020**, *132* (22), 8649–8656.

[12] Sung, J.; Nowak-Król, A.; Schlosser, F.; Fimmel, B.; Kim, W.; Kim, D.; Würthner, F. *J. Am. Chem. Soc.* **2016**, *138* (29), 9029–9032.

[13] M. H. Farag, A. I. Krylov, *J. Phys. Chem. C* **2018**, *122*, 25753–25763.

[14] Y. Hong, J. Kim, D. Kim, H. Kim, *J. Phys. Chem. A* **2021**, *125*, 875–884.

[15] Luzanov , A. V.; Casanova, D.; Feng, X.; Krylov, A. I. *J. Chem. Phys.* **2015**, 142, 224104.

[16] Shao, Y., Gan, Z., Epifanovsky, E., Gilbert, A. T. B., Wormit, M., Kussmann, J., Lange, A. W., Behn, A., Deng, J., Feng, X., Ghosh, D., Goldey, M., Horn, P. R., Jacobson, L. D., Kaliman, I., Khaliullin, R. Z., Kus, T., Landau, A., Liu, J., Proynov, E. I., Rhee, Y. M., Richard, R. M., Rohrdanz, M. A., Steele, R. P., Sundstrom, E. J., Woodcock, H. L., Zimmerman, P. M., Zuev, D., Albrecht, B., Alguire, E., Austin, B., Beran, G. J. O., Bernard, Y. A., Berquist, E., Brandhorst, K., Bravaya, K. B., Brown, S. T., Casanova, D., Chang, C., Chen, Y., Chien, S. H., Closser, K. D., Crittenden, D. L., Diedenhofen, M., DiStasio, R. A., Do, H., Dutoi, A. D., Edgar, R. G., Fatehi, S., Fusti-Molnar, L., Ghysels, A., Golubeva-Zadorozhnaya, A., Gomes, J., Hanson-Heine, M. W. D., Harbach, P. H. P., Hauser, A. W., Hohenstein, E. G., Holden, Z. C., Jagau, T., Ji, H., Kaduk, B., Khistyaev, K., Kim, J., Kim, J., King, R. A., Klunzinger, P., Kosenkov, D., Kowalczyk, T., Krauter, C. M., Lao, K. U., Laurent, A. D., Lawler, K. V., Levchenko, S. V., Lin, C. Y., Liu, F., Livshits, E., Lochan, R. C., Luenser, A., Manohar, P., Manzer, S. F., Mao, S., Mardirossian, N., Marenich, A. V., Maurer, S. A., Mayhall, N. J., Neuscamman, E., Oana, C. M., Olivares-Amaya, R., O'Neill, D. P., Parkhill, J. A., Perrine, T. M., Peverati, R., Prociuk, A., Rehn, D. R., Rosta, E., Russ, N. J., Sharada, S. M., Sharma, S., Small, D. W., Sodt, A., Stein, T., Stueck, D., Su, Y., Thom, A. J. W., Tsuchimochi, T., Vanovschi, V., Vogt, L., Vydrov, O., Wang, T., Watson, M. A., Wenzel, J., White, A., Williams, C. F., Yang, J., Yeganeh, S., Yost, S. R., You, Z., Zhang, I. Y., Zhang, X., Zhao, Y., Brooks, B. R., Chan, G. K. L., Chipman, D. M., Cramer, C. J., Goddard, W. A., Gordon, M. S., Hehre, W. J., Klamt, A., Schaefer, H. F., Schmidt, M. W., Sherrill, C. D., Truhlar, D. G., Warshel, A., Xu, X., Aspuru-Guzik, A., Baer, R., Bell, A. T., Besley, N. A., Chai, J., Dreuw, A., Dunietz, B. D., Furlani, T. R., Gwaltney, S. R., Hsu, C., Jung, Y., Kong, J., Lambrecht, D. S., Liang, W., Ochsenfeld, C., Rassolov, V. A., Slipchenko, L. V., Subotnik, J. E., Van Voorhis, T., Herbert, J. M., Krylov, A. I., Gill, P. M. W., Head-Gordon, M. *Mol. Phys.* **2015**, 113, 184−215.
